# Boron demanding tissues of *Brassica napus* express specific sets of functional Nodulin26‐like Intrinsic Proteins and BOR1 transporters

**DOI:** 10.1111/tpj.14428

**Published:** 2019-07-15

**Authors:** Till Arvid Diehn, Manuela Désirée Bienert, Benjamin Pommerrenig, Zhaojun Liu, Christoph Spitzer, Nadine Bernhardt, Jacqueline Fuge, Annett Bieber, Nicolas Richet, François Chaumont, Gerd Patrick Bienert

**Affiliations:** ^1^ Metalloid Transport, Department of Physiology and Cell Biology Leibniz Institute of Plant Genetics and Crop Plant Research (IPK) Gatersleben 06466 Germany; ^2^ Division of Plant Physiology University Kaiserslautern Kaiserslautern 67663 Germany; ^3^ Experimental Taxonomy, Genebank Department Leibniz Institute of Plant Genetics and Crop Plant Research (IPK) Gatersleben 06466 Germany; ^4^ Louvain Institute of Biomolecular Science and Technology UCLouvain Louvain‐la‐Neuve 1348 Belgium

**Keywords:** aquaporin, BOR transporter, boron, *Brassica napus*, Nodulin26‐like Intrinsic Protein, plant nutrition, transport

## Abstract

The sophisticated uptake and translocation regulation of the essential element boron (B) in plants is ensured by two transmembrane transporter families: the Nodulin26‐like Intrinsic Protein (NIP) and BOR transporter family. Though the agriculturally important crop *Brassica napus* is highly sensitive to B deficiency, and NIPs and BORs have been suggested to be responsible for B efficiency in this species, functional information of these transporter subfamilies is extremely rare. Here, we molecularly characterized the NIP and BOR1 transporter family in the European winter‐type cv.* Darmor‐PBY018*. Our transport assays in the heterologous oocyte and yeast expression systems as well as in growth complementation assays *in planta* demonstrated B transport activity of NIP5, NIP6, NIP7 and BOR1 isoforms. Moreover, we provided functional and quantitative evidence that also members of the NIP2, NIP3 and NIP4 groups facilitate the transport of B. A detailed B‐ and tissue‐dependent B‐transporter expression map was generated by quantitative polymerase chain reaction. We showed that NIP5 isoforms are highly upregulated under B‐deficient conditions in roots, but also in shoot tissues. Moreover, we detected transcripts of several B‐permeable NIPs from various groups in floral tissues that contribute to the B distribution within the highly B deficiency‐sensitive flowers.

## Significance Statement

Though *Brassica napus* plants are dependent on a continuous supply of the micronutrient boron, information on the orchestration of boron transport processes has been lacking. This study comprehensively analyzed a large set of boron transporters from the European winter‐type cv. *Darmor‐PBY018* at the molecular and functional level succeeding to unravel a cellular boron transporter network necessary to sustain boron nutritional demands during vegetative and reproductive growth.

## Introduction

Boron (B) is an essential element for vascular plants (Warington, 1923). Boron is the only nutrient, which, under physiological pH conditions, mainly occurs as a non‐charged molecule, namely boric acid. This is in equilibrium with its corresponding base, the borate ion [B(OH)_3_ + H_2_O ⇔ B(OH)_4_^−^ + H^+^; pk_a_ = 9.25]. When plants face B‐deficient growth conditions, a variety of irreversible deficiency symptoms can be observed, such as inhibited root and shoot elongation, modulated leaf expansion, deformed leaves, de‐differentiated vascular tissues, loss of fertility and flower abortion. The only described function of B at the molecular level is the bonding of two rhamnogalacturonan‐II monomers by borate esters in the pectin fraction of primary cell walls (O'Neill *et al*., 2001). This crosslinking is of crucial physiological significance because it sustains plant growth and development by simultaneously assuring stability and elasticity of the cell walls.

Growth of plants depends on a continuous external supply of B throughout the development, as this element cannot be remobilized in most plant species. Its long‐distance transport is highly connected to the transpiration stream leading to a quantitatively significant B flow towards fully developed and photosynthetically active source leaves. However, organs and tissues with a high demand for B are in fact mostly low transpiring such as young sink leaves, meristems, or the inflorescences. Therefore, plants face the challenge to permanently ensure B uptake from the soil and efficiently deliver B to low‐transpiring tissues (Brown and Shelp, 1997). To prevent irreversible tissue damages caused by spatiotemporal B deficiency – even under sufficient soil B bioavailability – plants require an efficient regulation of B fluxes.

This sophisticated B uptake and translocation regulation is ensured by two transmembrane transporter protein families. While Nodulin26‐like Intrinsic Proteins (NIPs) are passive and bidirectional membrane channels facilitating the diffusion of boric acid across membranes, BOR transporters are secondary active efflux transporters, transporting the borate anion (Miwa and Fujiwara, 2010; Parker and Boron, 2013). NIPs belong to the Major Intrinsic Protein family, which comprises distinct subfamilies being either essential for the regulation of the plant water homeostasis or for the facilitated diffusion of small solutes including metalloid acids (Bienert and Bienert, 2017; Roberts and Routray, 2017). BORs function mainly either in the active transfer of B to neighboring cell types or in the removal of B from cells into the apoplast to confer tolerance to high B (Miwa and Fujiwara, 2010). NIPs and BORs are often co‐expressed in the same cell but trafficked to opposite cell sides. Thereby, both transporter types function synergistically to optimize transcellular B fluxes, and to actively generate and maintain B gradients (Shimotohno *et al*., 2015).

This cooperative system ensures root B uptake, B root‐to‐shoot translocation and B loading into specific cells, tissues or the apoplast of different model and crop plant species (Takano *et al*., 2006; Miwa *et al*., 2007; Reid, 2007; Sutton *et al*., 2007; Tanaka *et al*., 2008; Kajikawa *et al*., 2011; Miwa *et al*., 2013; Chatterjee *et al*., 2014, 2017; Durbak *et al*., 2014; Hanaoka *et al*., 2014; Pallotta *et al*., 2014; Hua *et al*., 2016a; Routray *et al*., 2018; Shao *et al*., 2018). Additionally, BORs and NIPs have been demonstrated to be decisive factors determining plant B toxicity tolerance (Miwa and Fujiwara, 2010).

All cruciferous vegetables and crops, including various Brassica species, represent taxa that are extremely sensitive to B deficiency (Marschner, 2012). *Brassica napus* formed through interspecific crosses between the crops *Brassica rapa* (A genome) and *Brassica oleracea* (C genome). *Brassica napus* is widely cultivated and used worldwide for animal and human nutrition, as a catch and cover crop, and for biofuel production. *Brassica napus* exhibits detrimental and irreversible B deficiency symptoms, such ‘root rot’ or ‘flowering without seed setting’, and yield losses caused by temporally B‐limiting conditions are frequent (Wang *et al*., 2007). *Brassica napus* cultivars have soil B concentration requirements higher than 0.5 mg B (kg soil)^−1^, which exceed the concentrations in many agricultural soils (Shorrocks, 1997). In China, more than 33.3 million hectares of agricultural soils possess lower B concentrations (Xu *et al*., 2001). Therefore, studies addressing B efficiency in *B. napus* are of high agricultural and economic interest, but have been concentrated on a very limited number of genotypes (Xue *et al*., 1998; Stangoulis *et al*., 2001; Xu *et al*., 2001, 2002; Yang *et al*., 2013; Zhang *et al*., 2014a). *Brassica napus* cv. *Qingyou10* (*Q10*) and *Westar10* (*W10*) are B‐efficient and B‐inefficient cultivars, respectively, and served to study physiological, molecular and genetic factors influencing B efficiency (Zhao *et al*., 2012; Yang *et al*., 2013; Zhang *et al*., 2014a,b, 2017; Yuan *et al*., 2017; Zhou *et al*., 2017).

One major and one minor loci associating with yield‐related B efficiency traits encode *BnaA03.NIP5;1b* and *BnaC02.NIP5;1a*, respectively, which are sequence‐wise very similar to other NIP‐II type B channels from Arabidopsis, rice and maize (Hua *et al*., 2016a). Based on sequence and expression analyses, *BnaA03.NIP5;1b* was subsequently suggested to be the responsible gene in the B efficiency loci of cv. *Q10* (Xu *et al*., 2001, 2002; Zhao *et al*., 2008, 2012; Zhang *et al*., 2014a,b; Hua *et al*., 2016a,b). However, neither BnaA03.NIP5;1b nor BnaC02.NIP5;1a have been tested for their B permeability, although it is known that even highly homologous NIPs can possess different substrate selectivities (Zhao *et al*., 2009; Mitani‐Ueno *et al*., 2011). Another study suggested that transcript and protein abundance of *BnaC04.BOR1;1c* adds to the B deficiency tolerance of cv.* Q10* due to the fact that a knockdown mutant suffered from severe shoot and flower B deficiency symptoms when grown in B‐deficient growth conditions (Zhang *et al*., 2017; Chen *et al*., 2018).

Individual *BOR* and *NIP* transcript abundances in cv. *Q10* indeed exceeded those of the contrasting B deficiency‐sensitive genotype, cv.* W10*, both under B‐sufficient but also under B‐deficient growth conditions (Yuan *et al*., 2017; Chen *et al*., 2018). Functional assays assessing the transport selectivity of these channels and transporters are lacking. Comprehensive information on the B transporter set‐up of Asian *B. napus* cultivars is absent despite the fact that all until now identified highly B deficiency tolerant cultivars are of Asian origin (Zhang *et al*., 2014a; Pommerrenig *et al*., 2018). Moreover, studies dealing with potential B transporters of *B. napus* share the lack of functional analyses of actual BOR and NIP B transport activities. It is completely unknown which BnaNIPs facilitate the transmembrane diffusion of B and which ones not. BnaC04.BOR1;1c is the only isoform for which B transport activity has been suggested based on the analyses of *BnaC04.BOR1;1c* overexpressing and silenced plants (Zhang *et al*., 2017; Chen *et al*., 2018).

Therefore, in this study, we provided a genome‐wide comparison of all potentially B‐transporting BORs and NIPs that are encoded in the genomes of the Asian semi‐winter‐type *B. napus* cv.* Zhongshuang11* (*ZS11*; Sun *et al*., 2017) and the European winter‐type cv.* Darmor‐bzh* (Chalhoub *et al*., 2014). Moreover, we functionally characterized the NIP and BOR1 transporter family in cv. *Darmor‐PBY018*, which was described to be moderately B deficiency tolerant and which is genetically very close to the sequenced cv. *Darmor‐bzh* (Chalhoub *et al*., 2014; Schmutzer *et al*., 2015; Pommerrenig *et al*., 2018). Functional assays in the heterologous oocyte and yeast expression systems as well as *in planta* demonstrated a B transport activity for BnaNIP2, BnaNIP3, BnaNIP4, BnaNIP5, BnaNIP6, BnaNIP7 and BnaBOR1 isoforms. Additionally, quantitative polymerase chain reaction (qPCR) analyses identified the expression of several B‐permeable NIP isoforms in floral tissues of *Darmor‐PBY018*, which may distribute B within and to the highly B deficiency sensitive flowers.

## Results

### Comparison of AQPs and BORs between *Brassica napus* cultivars *Darmor‐bzh* and *ZS11*

**Figure 1:**
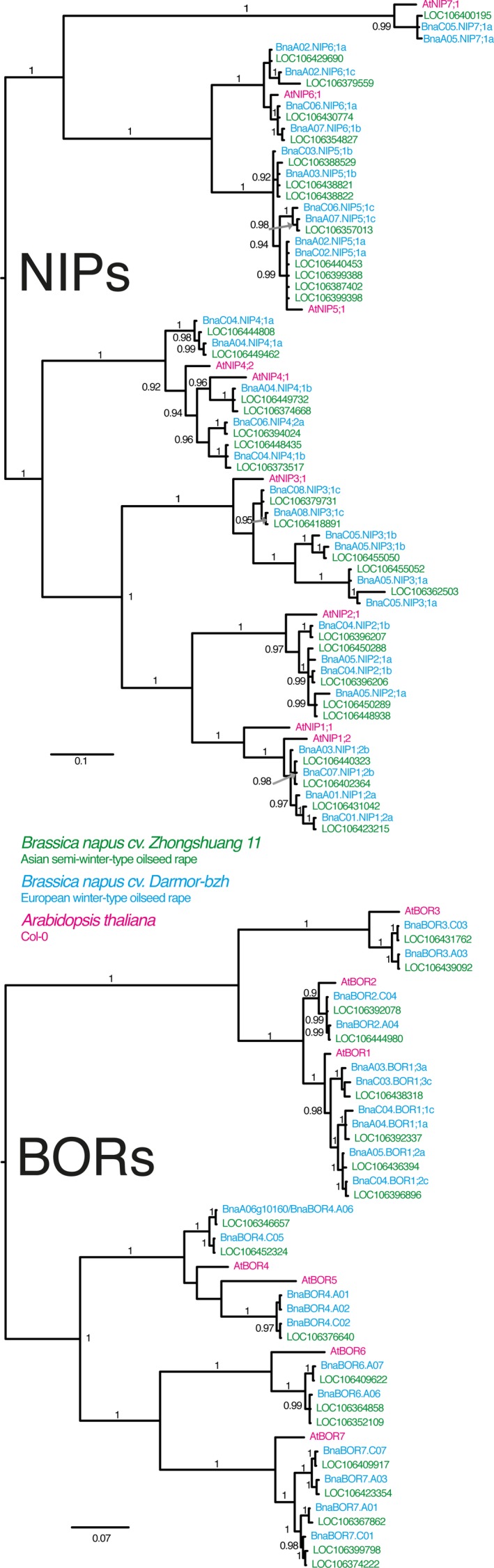
Phylogenetic analysis of the BOR transporter (BOR) and Nodulin26‐like Intrinsic Protein (NIP) families of two *Brassica napus* cultivars and Arabidopsis. Consensus trees derived from NIP (top panel) and BOR (lower panel) amino acid sequences from *Arabidopsis thaliana* (magenta) and the *B. napus* cultivars *Darmor‐bzh* (blue) and *Zhongshuang11* (green) using Bayesian phylogenetic inference. For the presentation of the tree midpoint rooting was applied. Numbers beside the nodes indicate the posterior probability values if larger than 0.9. For *Darmor‐bzh* NIPs and BORs the trivial names given by Yuan *et al*. (2017) and Chen *et al*. (2018) were used, while for *Zhongshuang11* NIPs and BORs the locus gene identifier name was used.

The genomic sequences of Arabidopsis *BOR* and *NIP* genes were used to identify homologous genes in the genomes of the *B. napus* Asian semi‐winter‐type cv.* ZS11* (Sun *et al*., 2017) and the European winter‐type cv.* Darmor‐bzh* (Chalhoub *et al*., 2014). Manual sequence assessment demonstrated that the BOR and NIP output coding sequences and exon/intron selections occasionally resulted in non‐satisfactory gene models. For such sequences, we were able to manually curate gene models encoding complete and more typical NIP and BOR features. In total, we identified 31 *NIP*‐ and 21 *BOR*‐ and 34 *NIP*‐ and 19 *BOR*‐ full‐length genes in *Darmor‐bzh* and *ZS11*, respectively (Figure 1).

Recently, two independent studies reported on the BOR and the aquaporin gene family of *Darmor‐bzh*, and identified 32 NIPs and 20 BORs, respectively (Yuan *et al*., 2017; Chen *et al*., 2018). We identified one additional full‐length *BOR* sequence in the *Darmor‐bzh* genome. In contrast to the study of Yuan *et al*. (2017), we excluded *BnaCnn_random.NIP4;1c* from further analyses as this sequence is not translated into a typical NIP4 protein isoform. While the NIP4 group amplified within the genomes of *B. oleracea* and *B. rapa* since the split from *Arabidopsis thaliana* (Diehn *et al*., 2015), rather a cutback was observed in *B. napus*. We hypothesize a reduction in the number of *NIP4* genes after the speciation of *B. napus* due to the fact that *BnaCnng65250* and *BnaC04g34460* that do not result in full‐length NIP4 sequences became pseudogenes in *Darmor‐bzh*.

Interestingly, the Chinese cv.* ZS11* has more NIPs but less BORs in its genome compared with the European cv. *Darmor‐bzh* (Figure 1). The overall protein sequence identity between the different homologous B transporters of the two cultivars is very high. This demonstrates that since the split of the European and Asian cultivars, the transport proteins, which are responsible for the B transport, did not diverge in terms of their coding sequences despite the different geographic, climatic, soil B availability and breeding‐histories. Due to in general lower soil B concentrations in Asian and particular Chinese compared with European rapeseed cultivation areas, semi‐winter‐type rapeseeds may have been evolutionarily forced to adapt to more B‐limiting conditions.

### Water permeability of BnaNIPs in Xenopus oocytes

**Figure 2:**
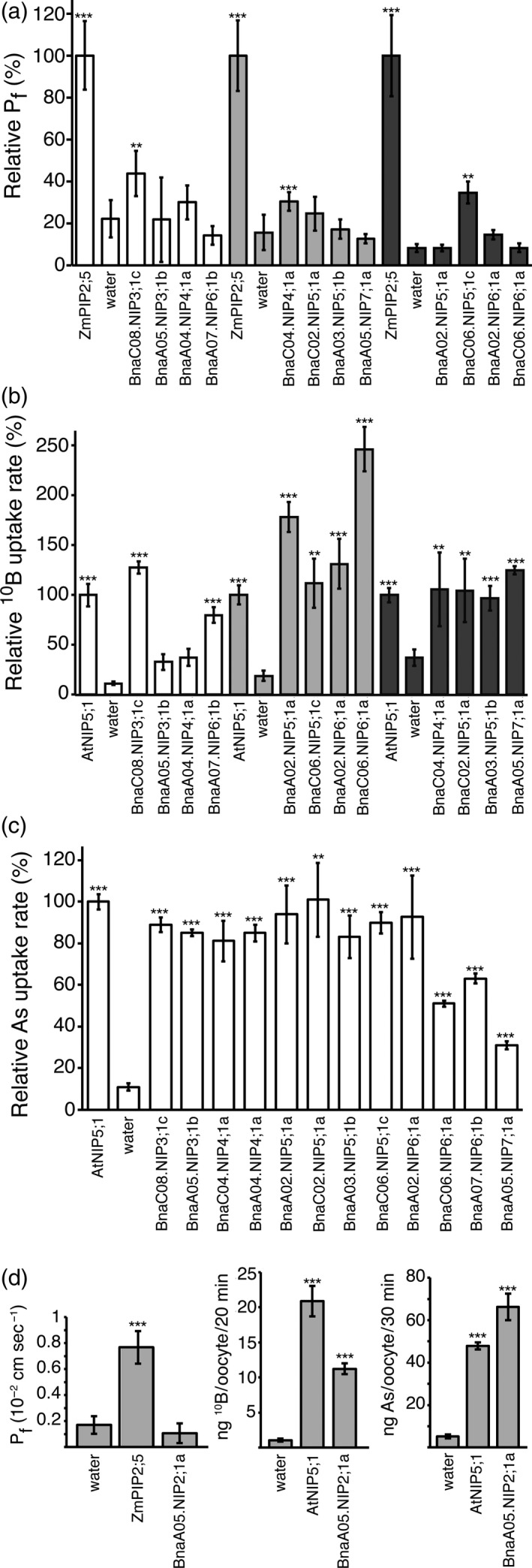
Transport capacities of *Brassica napus* Nodulin26‐like Intrinsic Protein (NIPs) determined in Xenopus oocytes. (a) Water transport ability of *B. napus* NIPs heterologously expressed in Xenopus oocytes. Xenopus oocytes were injected with 2 or 12.5 ng of *ZmPIP2;5* or *BnaNIP* cRNAs, respectively. P_f_ values were determined in a hypo‐osmotic swelling assay with Xenopus oocytes expressing NIPs or the highly water‐permeable positive control ZmPIP2;5. Oocytes injected with water were used as negative controls. Water transport measurements that were performed with oocytes deriving from the same frog are displayed with the same color (white, light gray or dark gray chart bars). Chart bars express the relative means (in %) of P_f_ measurements of 8–15 (controls) or 10–20 (BnaNIPs) oocytes, with respect to the P_f_ of the corresponding ZmPIP2;5 positive control oocytes. Error bars represent 95% CIs. (b) Permeability of BnaNIPs and AtNIP5;1 to boric acid in direct uptake assays. Oocytes expressing *AtNIP5;1* (positive control) or indicated *BnaNIPs* and the water‐injected negative control oocytes were exposed to a 5 mm ^10^boric acid containing Barth buffer solution for 20 min. B uptake assays that were performed with oocytes deriving from the same frog are displayed with the same color (white, light gray or dark gray chart bars). Chart bar values represent the relative means (in %) of ^10^B uptake rates per oocyte of four–five pools of oocytes (*n* = 9–11 oocytes per pool) per construct and with respect to the positive control AtNIP5;1. Error bars represent 95% CIs. ^10^B content of oocytes was determined by ICP‐MS analysis. (c) Arsenous acid uptake rates of Xenopus oocyte expressing *BnaNIPs* or *AtNIP5;1*. Oocytes expressing *AtNIP5;1* (positive control) or indicated *BnaNIPs* and water‐injected negative control oocytes were exposed to a 0.1 mm arsenite containing Barth buffer solution for 30 min. As content of oocytes was determined by ICP‐MS analysis. Chart bar values represent the relative means (in %) of As uptake rates per oocyte of three pools of oocytes (*n* = 8–10 oocytes per pool) per construct and with respect to the positive control AtNIP5;1. Error bars represent 95% CIs. (d) Water (left panel), boric acid (middle panel) and arsenous acid (right panel) uptake assays of oocytes expressing BnaA05.NIP2;1a, the indicated positive controls or being injected with water. Chart bar values represent the means of P_f_ values of 10–12 oocytes in the left panel, B uptake rates of three–five pools of oocytes (*n* = 10 oocytes per pool) in the middle panel, As uptake rates of three pools of oocytes (*n* = 10 oocytes per pool) in the right panel. Error bars represent 95% CIs. Asterisks indicate significant differences (*P < 0.05, *^*^ *P* < 0.01, *^**^ *P* < 0.001, *t*‐test) between the indicated NIP isoform and the water‐injected negative control oocytes. Water, boric acid and arsenous acid uptake assays have been twice or three times repeated per NIP isoform, with independent oocyte batches and with consistent results.

The water channel activities of BnaA05.NIP2;1a, BnaC08.NIP3;1c, BnaA05.NIP3;1b, BnaC04.NIP4;1a, BnaA04.NIP4;1a, BnaA02.NIP5;1a, BnaC02.NIP5;1a, BnaA03.NIP5;1b, BnaC06.NIP5;1c, BnaC06.NIP6;1a, BnaA02.NIP6;1a, BnaA07.NIP6;1b and BnaA05.NIP7;1a were tested by heterologous expression in *Xenopus laevis* oocytes. High osmotic water permeability coefficient (P_f_) values were obtained for oocytes expressing the positive control ZmPIP2;5 (Chaumont *et al*., 2000). Only oocytes expressing BnaC08.NIP3;1c, BnaC04.NIP4;1a and BnaC06.NIP5;1c showed a significant increase in P_f_ compared with water‐injected control oocytes (Figure 2a). The P_f_ values of these three isoforms is, however, low compared with ZmPIP2;5, which is a physiologically important and typical water channel.

### *Brassica napus* NIP2, NIP3, NIP4, NIP5, NIP6 and NIP7 isoforms are functional metalloidoporins when expressed in Xenopus oocytes

To test the boric acid transport ability of BnaNIP proteins, a direct B uptake assay into oocytes was performed. To this aim, water‐injected negative control oocytes or oocytes expressing the different BnaNIPs or the positive control AtNIP5;1 were exposed to a buffer containing 5 mm ^10^boric acid for 20 min. Significant B uptake levels were detected for the positive control AtNIP5;1, as well as for BnaA05.NIP2;1a, BnaC08.NIP3;1c, BnaC04.NIP4;1a, BnaA02.NIP5;1a, BnaC02.NIP5;1a, BnaA03.NIP5;1b, BnaC06.NIP5;1c, BnaC06.NIP6;1a, BnaA02.NIP6;1a, BnaA07.NIP6;1b and BnaA05.NIP7;1a, but not for BnaA05.NIP3;1b and BnaA04.NIP4;1a (Figure 2b,d). BnaA05.NIP3;1b and BnaA04.NIP4;1a represent interesting candidates to unravel the so far unknown metalloid substrate specificity of these NIP subgroups in further studies.

Previous studies demonstrated that boric acid permeability goes along with arsenous acid permeability in NIP channel proteins (Mitani‐Ueno *et al*., 2011). To determine the arsenous acid transport activity of BnaNIPs, oocytes expressing them were exposed to a buffer solution containing 0.1 mm NaAsO_2_ for 30 min. AtNIP5;1 was used as a positive control. Arsenic permeability was detected for AtNIP5;1 and all tested BnaNIPs (Figure 2c,d), suggesting that all channel proteins were correctly folded and localized to the plasma membrane. This further demonstrated that an inexistent water permeability does not result from a problem in protein expression or plasma membrane targeting in oocytes.

### *Brassica napus* NIP4, NIP5, NIP6 and NIP7 rescue the B deficiency phenotype of the Arabidopsis *nip5;1* knockout mutant

**Figure 3:**
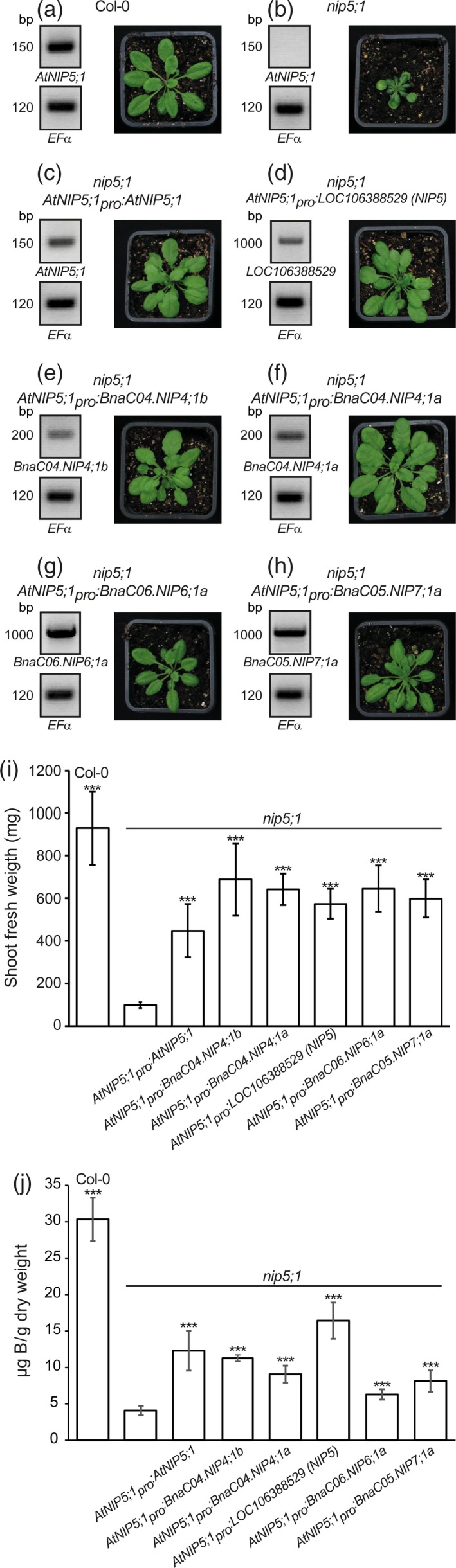
Expression of *Brassica napus* NIP4, NIP5, NIP6 and NIP7 isoforms rescue the B deficiency phenotype of Arabidopsis *nip5;1* knockout mutants. (a–h) Representative shoot growth of the wild‐type (Col‐0) (a), the *Atnip5;1* knockout line (b), or *Atnip5;1* knockout lines transformed with the indicated different *AtNIP5;1*_*pro*_ *:BnaNIPs* (d–h) or with *AtNIP5;1*_*pro*_ *:AtNIP5;1* (c) as well as the reverse transcriptase‐polymerase chain reaction (RT‐PCR)‐based confirmation of the expression of the indicated *NIP* transgene in roots of the displayed plant line under B‐deficient conditions. Expression of *EF*α was used as control. (i) Shoot fresh weight quantification of the indicated Arabidopsis genotypes grown under standard greenhouse soil substrate conditions in which *Atnip5;1* knockout lines show obvious B deficiency symptoms and growth retardation. (j) B concentration of above‐mentioned (i) Arabidopsis rosettes (*n* = 6–7). Error bars represent SD values. Significance was calculated using *t‐*test against the basal B concentration of the *Atnip5;1* knockout line (***P < 0.001).

To assess B transport ability *in planta*, we expressed representative BnaNIPs from the NIP4, NIP5, NIP6 and NIP7 group under the control of the *AtNIP5;1* promoter (*AtNIP5;1*_*pro*_) in the *Atnip5;1* knockout background (Takano *et al*., 2006). In contrast to Col‐0 wild‐type plants, *Atnip5;1* knockouts suffered from B deficiency under standard greenhouse soil substrate conditions [0.4 mg B (kg soil)^−1^; Figure 3a–c]. Independent *Atnip5;1* knockout lines, transformed with the positive control *AtNIP5;1*, or *BnaC04.NIP4;1b*,* BnaC04.NIP4;1a*,* BnaLOC106388529 (NIP5)*,* BnaC06.NIP6;1a* or *BnaC05.NIP7;1a* were grown on B‐deficient (0.2 μm B) MS‐agar media, where the *AtNIP5;1*_*pro*_ is active and all the lines expressed the corresponding *BnaNIP* isoforms as verified by reverse transcription (RT)‐PCR on root cDNA (Figure 3a–h). As shown in Figure 3(a–j), the *BnaNIP* expressing lines of the T3 generation grew significantly better, reached significantly heavier shoot fresh weights and had a significantly higher B uptake capacity, indicated by the higher shoot B concentrations, than the *nip5;1* knockouts, demonstrating their functionality and ability to facilitate the uptake of B into plant roots. However, these lines still displayed weak B deficiency symptoms, such as cupped downward leaves and an impaired fertility when grown under B‐sufficient conditions. This was also the case for the lines expressing *AtNIP5;1*_*pro*_ *:AtNIP5;1* constructs that did not fully restore the wild‐type growth behavior under B‐sufficient conditions and depended on an extra‐fertilization of B during the reproductive stage to generate fertile flowers.

### All six BnaBOR1 isoforms from *Brassica napus* are functional B efflux transporters when expressed in yeast

**Figure 4:**
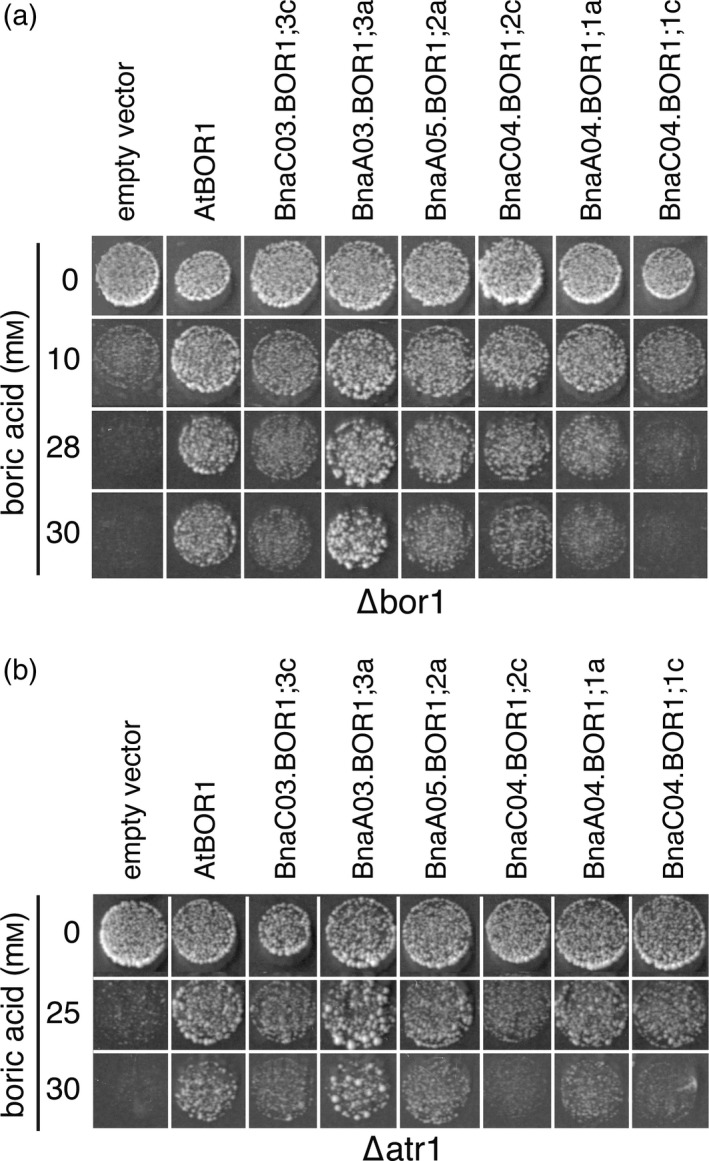
Boric acid export activity of *Brassica napus* BOR1s in ∆*atr1* and ∆*bor1* yeast cells. Boric acid toxicity growth assays under high‐B conditions in the *∆bor1* (a) and *∆atr1* (b) mutant yeast strains expressing the indicated BOR1 isoforms. Cultures of mutant yeast cells transformed with the empty vector *pYeDP60u* or *pYeDP60u* carrying *AtBOR1* or the indicated *BnaBOR1* cDNAs were diluted in sterile water and spotted on medium containing the indicated concentrations of boric acid. Growth behavior and survival rates of the different transformants were recorded after 5–10 days at 30°C and are shown for yeasts spotted at an OD_600_ of 0.01. All yeast growth assays were performed at least twice with independent transformation events and with consistent results. Displayed images in (a) and (b) represent groups of sub‐images assembled from different growth plates and conditions.

*Saccharomyces cerevisiae* mutants lacking either Bor1p or Atr1p, two B efflux transporters involved in the B detoxification system, are highly sensitive to high B conditions (Takano *et al*., 2007; Kaya *et al*., 2009). To test whether BnaBOR1s are functional B exporters, all six BOR1 isoforms were expressed individually in both yeast mutants, and a toxicity growth assay on media supplemented with increasing amounts of boric acid was performed. *∆bor1* yeast mutants expressing BnaBOR1s grew better than those carrying the empty vector on medium supplemented with > 10 mm boric acid (Figure 4a). No growth differences were observed when yeast cells were grown on medium without addition of B. The growth of the yeast expressing BnaA03.BOR1;3a, BnaC04.BOR1;2c, BnaA05.BOR1;2a and BnaA04.BOR1;1a was similar to the positive control expressing AtBOR1, while it was slightly weaker for BnaC03.BOR1;3c and BnaC04.BOR1;1c expressing yeast. ∆*atr1* mutant cells expressing the different BOR1s had also a rescued growth at boric acid concentrations in the medium higher than 25 mm, while growth of the strain with the empty vector (negative) control was not detected (Figure 4b). In both yeast mutant strains, cells expressing BnaA03.BOR1;3a showed the best growth complementation, and their growth was even more vigorous compared with cells expressing the positive control AtBOR1.

### *Brassica napus* NIP and BOR1 transporter expression is tissue‐specific and responds to a changing boron nutritional status in vegetative and reproductive organs

To test where and when BnaBOR1s and BnaNIPs potentially play a role in B uptake and translocation, tissue‐, developmental‐ and B‐dependent expression maps for *BOR1* and *NIP* transporter genes were generated based on the premise that we succeeded to design gene‐specific primer pairs or that such primer pairs were available. qPCR was performed for six *BOR1s* and 23 *NIPs* on RNA samples extracted from *B. napus* cv.* Darmor‐PBY018* plants during different developmental stages, ranging from the early vegetative stage to the onset of flowering.

Plants have been cultivated either in soil‐substrate in the greenhouse, under near‐field conditions or hydroponically to have a controlled access to the root system.

**Figure 5:**
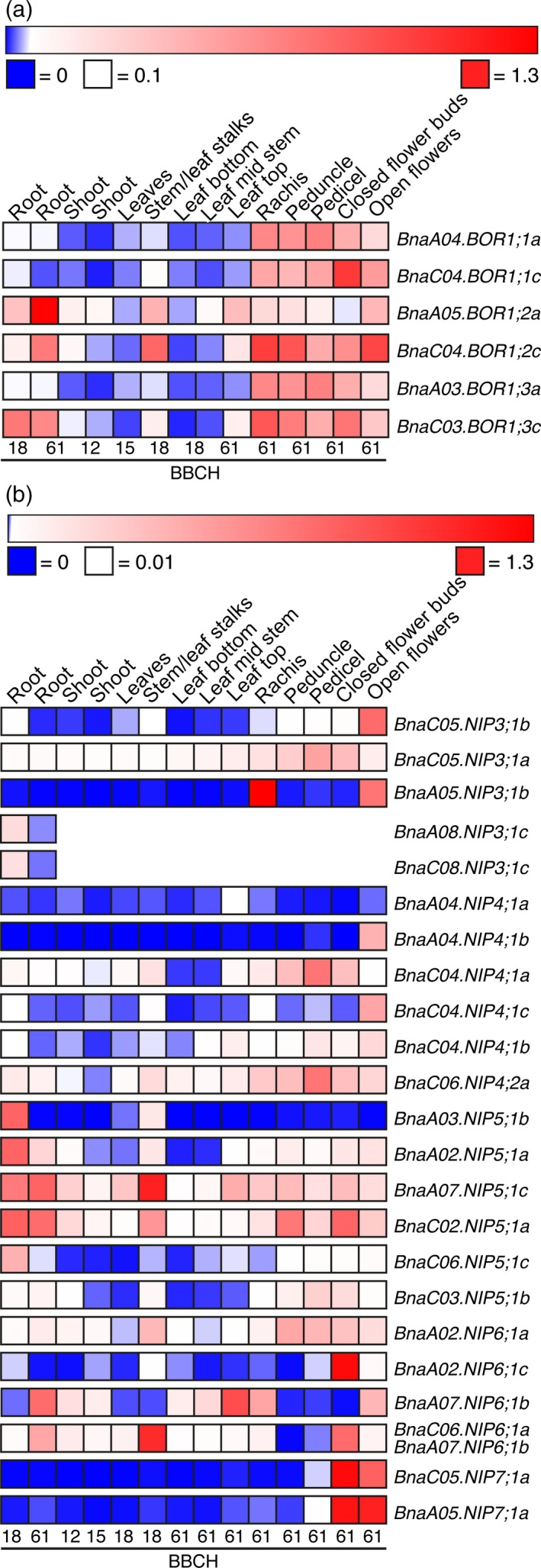
Tissue‐specific and developmental‐dependent expression profile of *BnaNIP* and *BnaBOR1* genes in cv. *PBY018*. Relative expressions of *BOR1* (a) and *NIP* (b) genes were determined by quantitative polymerase chain reaction (qPCR) and calculated using a gene expression normalization factor for each tissue sample based on the geometric mean of three reference genes (see Experimental procedures). To calculate the ∆Ct for each *NIP* or *BOR1* gene of interest, the tissue that resulted in the lowest Ct value was chosen as a reference. Relative expression values are displayed in a heat map representation that was generated using the Morpheus software. The color scheme describes the relative expression of each *NIP* or *BOR1* gene ranking from 0 (blue), over 0.01 (white) to 1.3 (red) in (a) or from 0 (blue), over 0.1 (white) to 2.1 (red) in (b). Expression was determined at different developmental stages according to the BBCH (‘Biologische Bundesanstalt, Bundessortenamt und CHemische Industrie’) stage definition (Lancashire *et al*., 1991) of plants that were grown on B‐sufficient conditions as described in detail in the Experimental procedures (Supporting Experimental procedures Methods S1). BBCH 12, 15 and 18 (principal growth stage 1: leaf development 1): 2, 5 and 8 leaves are unfolded, respectively; BBCH 61 (principal growth stage 6: flowering): 10% of flowers on main raceme open, main raceme elongating.

*BnaA04.BOR1;1a*,* BnaC04.BOR1;1c*,* BnaA05.BOR1;2a*,* BnaC04.BOR1;2c*,* BnaA03.BOR1;3a* and *BnaC03.BOR1;3c* were strongly expressed in flower tissues and the flower‐bearing stem portion of the inflorescence (rachis) during the reproductive growth stage (Figure 5a). The transcript abundance of *BnaA05.BOR1;2*a, BnaC04*.BOR1;2c* and *BnaC03.BOR1;3c* was also high in roots during the vegetative and reproductive stages. The expression of *BOR1s* was low in leaves, but higher in the stem and leaf stalks of the corresponding leaves.

**Figure 6:**
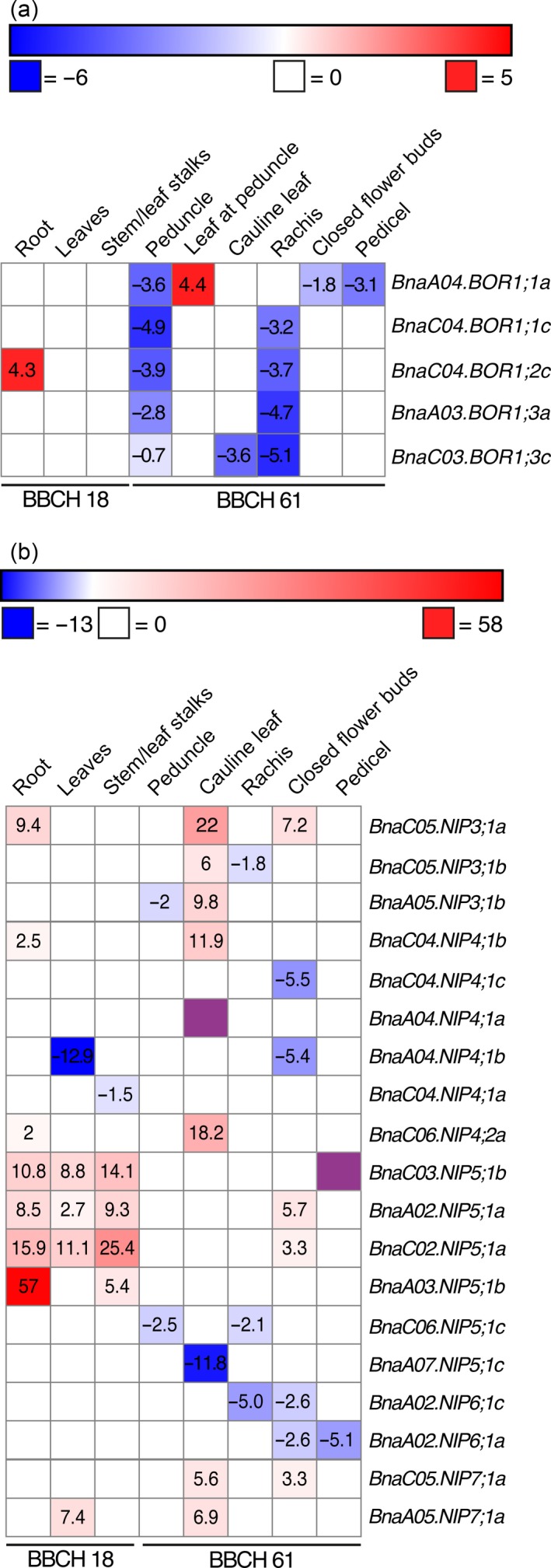
Tissue‐ and developmental‐specific expression profile of *BnaNIP* and *BnaBOR1* genes in *Brassica napus* cv.* PBY018* in dependence of the boron availability. Relative expression of *BOR1* (a) and *NIP* (b) genes under B‐sufficient and B‐deficient conditions were determined by quantitative polymerase chain reaction (qPCR). Significant (*P* < 0.05) fold up‐ or fold downregulation under B‐deficient compared with B‐sufficient growth conditions are displayed. The heat map of expression profiles was generated using Morpheus software. The color scheme describes the range of up‐ or downregulation of the indicated NIPs ranking from a 13‐fold down‐ (blue) to a 58‐fold upregulation (red). Purple‐colored squares indicate a more than > 1000‐fold downregulation, which can be interpreted as a switch‐off of the expression or an increased mRNA degradation. Expression was determined at different developmental stages according to the BBCH (‘Biologische Bundesanstalt, Bundessortenamt und CHemische Industrie’) stage definition (Lancashire *et al*., 1991). BBCH 12, 15 and 18 (principal growth stage 1: leaf development 1): 2, 5 and 8 leaves are unfolded, respectively; BBCH 61 (principal growth stage 6: flowering): 10% of flowers on main raceme open; main raceme elongating.

Interestingly, *BOR1* expression was, in general, downregulated in the rachis and the peduncle under B‐deficient conditions. *BnaC04.BOR1;2c* and *BnaA04.BOR1;1a* were the only BOR1s that showed a significant upregulation under B‐deficient conditions in young roots and leaves at the peduncle, respectively (Figure 6a).

*BnaA05.NIP3;1a* and *BnaA05.NIP3;1b* as well as *BnaC05.NIP3;1a* and *BnaC05.NIP3;1b* are placed in tandems within the genome, respectively. No specific primers for the genomic tandem partner *BnaA05.NIP3;1a* of *BnaA05.NIP3;1b* could be obtained. At the beginning of flowering when about 10% of flowers on the main and elongating raceme are open (BBCH61), *BnaA05.NIP3;1b* was specifically expressed in the rachis and open flowers, but interestingly not in closed flower buds and all other tested tissues. *BnaC05.NIP3;1a* was the most widespread transcribed member of the *NIP3* group as little expression was detected in all assayed tissues (Figure 5b). *BnaC05.NIP3;1b* and *BnaC05.NIP3;1a* displayed a differential expression, as a substantial expression of *BnaC05.NIP3;1b* was only detected in open flowers. Expression of *BnaA08.NIP3;1c* and *BnaC08.NIP3;1c* that show high sequence identity and represent a syntenic *NIP3* gene pair of the A and C genomes were quantified and detected in roots but not in other tissues, as the primer pair became unspecific therein (Figure 5b). Under B‐deficient conditions, *BnaC05.NIP3;1a* was strongly upregulated in roots and closed flowers, while *BnaC05.NIP3;1b* was downregulated in the rachis. Expression of *NIP4* group genes was very marginal in vegetative tissues. With the exception of *BnaA04.NIP4;1a*, all other *NIP4s* were expressed in open flowers. Additionally, *BnaC04.NIP4;1a* and *BnaC06.NIP4;2a* were clearly detected in the peduncle, the rachis, pedicels and closed flower buds. *BnaA04.NIP4;1b* was specifically expressed in open flowers. While under B‐deficient conditions *BnaC06.NIP4;2a* and *BnaC04.NIP4;1b* were twofold upregulated in young roots, *BnaC04.NIP4;1c* and *BnaA04.NIP4;1b* were fivefold downregulated in closed flower buds. Interestingly, a strong upregulation of three and two *NIP3* and *NIP4* isoforms, respectively, was observed under B‐deficient conditions in cauline leaves of flowering plants, while *BnaA04.NIP4;1a* was strongly downregulated.

With the exception of *BnaC03.NIP5;1b*, all *NIP5* genes were strongly expressed in roots of different developmental stages. *BnaA07.NIP5;1c* and *BnaC02.NIP5;1a* transcript were detected in all assayed tissues. Low expression of *BnaA02.NIP5;1a* and *BnaA03.NIP5;1b* was also detected in shoot parts such as the stem and leaf stalks. With the exception of *BnaC06.NIP5;1c* and *BnaA07.NIP5;1c*, all other *NIP5;1* genes were strongly upregulated (between 2.7‐ and 57‐fold) in roots, leaves, leaf stalks and the stem in B‐deficient conditions. The syntenic *NIP5* pair, *BnaA02.NIP5;1a* and *BnaC02.NIP5;1a*, was five and three times upregulated in closed flower buds under B‐deficient conditions.

The expression pattern of the four *NIP6* genes was quite heterogeneous (Figure 5b). The highest transcript abundances of *BnaA07.NIP6;1b*,* BnaA02.NIP6;1a*,* BnaA02.NIP6;1c* and *BnaC06.NIP6;1a* were detected in young leaves of the inflorescence, peduncles, closed flower buds and stem/leaf stalks, respectively. Interestingly, under B‐limiting conditions, no upregulation of any of the assayed *NIP6* genes was detected. In contrast, expression was downregulated in floral tissues. *NIP7* genes represented the highest expressed *NIP* genes. Their expression was restricted to closed‐ and open flowers. No significant transcript changes were observed for any of the *NIP* genes in open flowers and bottom‐ and mid leaves during the reproductive growth stage.

### *BnaNIP5;1* promoter activities are strongly upregulated in Arabidopsis under B‐limiting conditions

**Figure 7:**
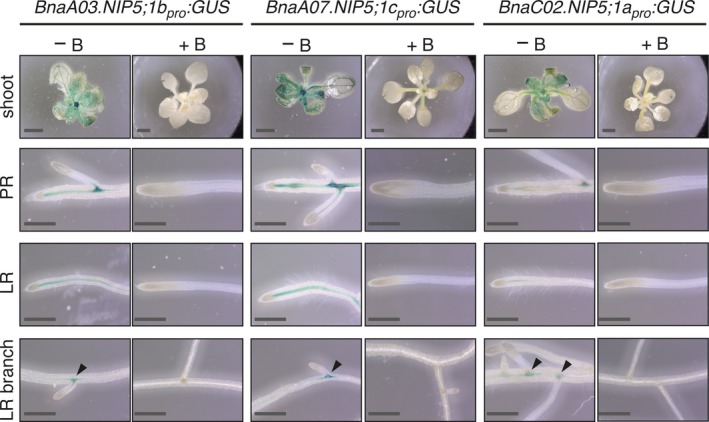
*BnaNIP5;1* promoters are upregulated in Arabidopsis roots and shoots under B limitation. B‐dependent *BnaNIP5;1* promoter activity in Arabidopsis roots and shoots visualized by promoter GUS analysis. Homozygous T3 *BnaNIP5;1*_*pro*_ *:GUS* transgenic plant lines were grown for 7 days without the supply of any boric acid and then transferred to medium containing 0.1 (B‐deficient) or 100 (B‐sufficient) μm boric acid. After 9 days, GUS staining was performed for 16 h. Representative shoots, primary roots (PR), lateral roots (LR) and lateral root branch point (LR branch) pictures are displayed. Black arrowheads indicate GUS signal at sites where lateral roots have emerged. For each *BnaNIP5;1*_*pro*_ *:GUS* construct two independent transgenic lines have been assessed with identical results. Scale bars: shoots = 2 mm and roots = 0.5 mm.

According to the qPCR results, BnaNIP5 genes were most responsive to B‐deficient conditions. We further elucidated the B‐dependent expression of *BnaNIP5* genes in the T3 generation of transgenic Arabidopsis Col‐0 plants expressing the β‐glucuronidase (*GUS*) gene under the control of three different *BnaNIP5* promoters (*BnaC02.NIP5;1a*_*pro*_ *:GUS*,* BnaA07.NIP5;1c*_*pro*_ *:GUS*,* BnaA03.NIP5;1b*_*pro*_ *:GUS*). GUS signals were detected after 4 (Figure S3a), 8 (Figure S3b) and 16 (Figure 7) h of incubation times to exclude experimentally caused artificial localizations of the GUS signals. GUS activity was consistently higher in all transgenic plants that have been transferred at 7 days after germination to MS‐medium with low B supply (0.1 μm B) compared with plants transferred to a high B supply (100 μm B; Figures 7 and S3). Plants grown on 100 μm B did not display any obvious promoter‐specific GUS signal. Interestingly, GUS activity was strongly enhanced in the shoots of all *BnaNIP5;1*_*pro*_ *:GUS*‐expressing transformants upon B‐deficient conditions (Figures 7 and S3). The GUS signal was located to the vasculature of the leaves and in the shoot apical meristem. Likewise, an upregulation of the corresponding *BnaNIP5* genes was detected by qPCR analysis in shoots of young rapeseed plants (Figure 6b). An obvious upregulation of *NIP5* genes of *B. napus* in shoot tissue contrasts with the *AtNIP5;1* expression, which is primarily detected in roots upon B deficiency but not in the shoots (Takano *et al*., 2006). An increased GUS activity in a few, but not all, primary and lateral roots was observed for *BnaC02.NIP5;1a*_*pro*_ *:GUS*,* BnaA07.NIP5;1c*_*pro*_ *:GUS* and *BnaA03.NIP5;1b*_*pro*_ *:GUS* expressing plants under B‐deficient conditions (Figure 7). The GUS signal was mainly detected in the vasculature cylinder close to primary and lateral root tips. Interestingly, strong GUS activity was frequently visible at sites of the vascular cylinder where lateral roots just had emerged. Further away from these sites the GUS activity fainted out. In all cases, a prolonged GUS staining time (from 4 via 8 to 16 h) resulted in a consistent and gradual increase in the detected GUS signal intensity but not in a different GUS localization pattern (Figures 7 and S3a,b).

## Discussion

Despite having a short speciation time of about 7500 years, *B. napus* displays an impressive genetic diversity (Chalhoub *et al*., 2014). This is due to the fact that this allopolyploid species formed probably several times independently through interspecific crosses between *B. rapa* and *B. oleracea*. Thereafter, several genetically distinct variants have been developed in adaption to geographic, environmental and nutritional demands, and according to breeding objectives. This resulted in European winter‐, Asian semi‐winter‐, Canadian‐, Australian‐, Asian‐ and European spring‐type cultivars. All *B. napus* types have a high demand for B in common. European rapeseeds have been cultivated from the Middle Age onwards and spread throughout the world. Since the 1970s, ‘double low’ alleles from Canadian and Polish genotypes have been introgressed into European cultivars. Cv.* Darmor‐bzh* shares this history (Chalhoub *et al*., 2014). Cv. *ZS11* is a Chinese semi‐winter‐type ‘double low’ variety. Chinese rapeseeds probably derive from European cultivars. Subsequently, they were modified by introgression of *B. rapa* cultivars that had been cultivated for more than 1000 years in Asia. These introgression events amplified their genetic diversity. From the 1970s/1980s, European ‘double low’ cultivars have been introgressed into the Chinese *B. napus* gene pool. Cv.* ZS11* belongs to this latter pool of cultivars. Due to their separate breeding history, adaptions to different soils and climates (Chalhoub *et al*., 2014; Sun *et al*., 2017), we expected a different B transporter composition between *ZS11* and *Darmor‐bzh*, but found a very similar configuration and a high protein sequence identity of transporters encoded by syntenic gene pairs. In future, comparison of the herein described B transporters with those of Asian cultivars existing prior to the introgression of ‘double low’ traits from non‐Asian cultivars will help to understand evolution of B transport during domestication of *B. napus* and to identify B efficiency transport traits that can be used for the breeding of modern elite lines.

To shed light into the scarcely elucidated molecular functions and regulations of B transporters of rapeseed, we cloned a large set of selected NIPs (from different subfamilies) and BOR1s, tested their transport selectivity and characterized them at the molecular level.

### BOR characteristics

Using the *∆atr1* and *∆bor1* yeast mutant strains, we demonstrated that all six BOR1s of rapeseed are functional B transporters. The fact that rapeseed kept all six BOR1 isoforms, which have formed by genome multiplications since the evolutionary split from Arabidopsis, underlines their importance for a tightly operated B transport regulation. We then investigated the tissue‐ and B‐dependent expression to understand when and where BOR1s may play a role in B transport.

Under B‐deficient growth conditions, *BnaC04.BOR1;2c* and *BnaA04.BOR1;1a* were significantly upregulated in roots and leaves at the peduncle. Our expression map significantly advanced the state‐of‐the‐art knowledge on BOR1 expression patterns, especially in floral tissues. Besides their root expression, all *BOR1s* showed highest expression in the rachis and in open flowers, suggesting that these isoforms are particularly important to deliver B to these highly B deficiency sensitive organs. In contrast to Chen *et al*. (2018), no B deficiency‐mediated upregulation of *BnaC04.BOR1;1c*,* BnaA05.BOR1;2a* and *BnaA03.BOR1;3a* was detected, neither in the roots, the shoots nor the diverse floral tissues. This indicates that different rapeseed cultivars developed a cultivar‐specific BOR1 expression pattern, which may result from the adaptation to different environmental demands. The quantified constitutive high expression of *BOR1s* might help to constantly ensure B delivery to flowers even under suddenly occurring B‐limiting periods when a *de novo* translation might be too late to prevent detrimental effects.

### NIP characteristics

Experimental data on NIPs of different plant species suggest that they are either impermeable or only weakly permeable to water (Roberts and Routray, 2017). This is in agreement with our results, namely that only BnaC08.NIP3;1c, BnaC04.NIP4;1a and BnaC06.NIP5;1c showed a slightly increased water permeability. The P_f_ values of the oocytes expressing these three isoforms are, however, very low compared with that of oocytes expressing typical ZmPIP2;5 water channel.

Performing B isotope‐discrimination uptake assays in oocytes, we detected significant B permeability for all tested NIP2–NIP7 isoforms, except for BnaA05.NIP3;1b and BnaA04.NIP4;1a. Our quantitative assays provided functional evidence that, in addition to members of the NIP5, NIP6 and NIP7 groups, also members of the NIP2, NIP3 and NIP4 groups facilitate the transmembrane diffusion of B. These results indicate that also the latter isoforms that have not been assayed for B transport before have an impact on the B nutritional status of rapeseed plants, and have to be considered in further studies dealing with B transport processes in plants. Interestingly, all assayed NIP2–NIP7 isoforms were permeable to As, also BnaA05.NIP3;1b and BnaA04.NIP4;1a, which did not significantly increase the uptake of B into oocytes. This observation is in agreement with other studies suggesting that As permeability is widespread amongst NIP aquaporin subgroups, and that the restrictions for As passage through NIP channels appear to be lower than those for other metalloids such as B (Zhao *et al*., 2009; Mitani‐Ueno *et al*., 2011; Xu *et al*., 2015). Interestingly, permeability to As had not been demonstrated for NIP2s and NIP4s, previously.

Transport assays with AtNIP7;1 expressed in Xenopus oocytes showed extremely low B transport rates due to a suggested gating function of a conserved tyrosine in helix 2 (Li *et al*., 2011). In contrast, BnaA05.NIP7;1a exhibited significantly higher B and As transport rates compared with the negative controls, providing evidence for the activity of the native NIP7 isoforms also in biological membranes despite the presence of the Tyr81 in helix 2. Together, our direct transport assays in oocytes suggest that the tested NIP2–NIP7 group isoforms are functional B and As channels in rapeseed, and have therefore an impact on the distribution of B and As in this high‐B‐demanding crop.

We experimentally confirmed the B transport ability of BnaNIPs in *in planta* growth complementation assays using *Atnip5;1* knockout lines expressing different *BnaNIPs* under the control of the *AtNIP5;1* promoter. Obvious growth defects and B deficiency symptoms of the *Atnip5;1* mutants were not observed during the vegetative growth stage of *AtNIP5;1*_*pro*_ *:BnaC04.NIP4;1b*,* AtNIP5;1*_*pro*_ *:BnaC04.NIP4;1a*,* AtNIP5;1*_*pro*_ *:BnaLOC106388529 (NIP5)*,* AtNIP5;1*_*pro*_ *:BnaC06.NIP6;1a* or *AtNIP5;1*_*pro*_ *:BnaC05.NIP7;1a* expressing transformants. These results strikingly demonstrated that the assayed BnaNIP4, BnaNIP5, BnaNIP6 and BnaNIP7 isoforms are also functional metalloidoporins in plants. Unexpectedly, all NIP transformants, including the *AtNIP5;1*_*pro*_ *:AtNIP5;1* ones, had lower shoot fresh weights than the Col‐0 wild‐type plants and depended on a surplus of B during the vegetative growth stage to be able to develop fertile flowers even under standard growth conditions. For NIP4 and NIP7 transformants, one may speculate that a lacking polar localization of these isoforms was responsible for the incomplete recovery of the wild‐type growth behavior, as only correctly polar‐localized Arabidopsis NIPs, such as AtNIP5;1 or AtNIP6;1, totally complement *Atnip5;1* mutants (Wang *et al*., 2017). The *AtNIP5;1*_*pro*_ fragment that we used differs slightly from the *AtNIP5;1*_*pro*_ constructs that were used in earlier studies due to cloning specification (Takano *et al*., 2006; Wang *et al*., 2017). Additionally, our constructs contained the coding‐ and not the genomic *AtNIP5;1* sequence for expression. Why the *NIP5* transformants in this study did not fully rescue the mutant phenotype remains to be elucidated in future. We speculate that this might be due to either missing promoter or enhancer elements located upstream and not integrated in our promoter, or harsher B‐limiting soil conditions than used in Takano *et al*. (2006).

In contrast to previous studies on B transporter expression patterns in plants, we particularly focused on the expression of B transporters under B‐deficient and B‐sufficient conditions in floral tissues. This was possible as we successfully managed to set up soil‐substrate‐based growth conditions in which plants can grow phenotypically identical until the flowering stage (BBCH60) under B‐sufficient and B‐deficient conditions. At this growth stage, the first flowers developed normally without any obvious B deficiency phenotypes, though the plants possessed intrinsic B levels, which are typical for severely B‐deficient rapeseed plants (Figure S1). Thereupon, from BBCH61 onwards, typical B deficiency symptoms appeared and finally resulted in the ‘flowering without seed setting’ syndrome. This growth set‐up allowed us to investigate in detail the B‐dependent expression of B transporters under controlled and reproducible B‐deficient conditions during the onset of flowering, when the inflorescences are particular susceptible to B deficiency. As flowering is the developmental stage that is most prone to B deficiency and subsequent yield losses, it is highly important to understand how B logistics are regulated there and to spot transport bottlenecks, firstly in flowers themselves but also in tissues supplying nutrients to the flowers, such as the rachis, peduncles and pedicels. This knowledge provides the basis to potentially enhance transport efficiency in rapeseed and prevent B deficiency caused yield losses. Using this growth set‐up, we determined tissue‐ and B‐dependent expression patterns at ≥ BBCH60 to understand when and where NIPs might play a role in floral B transport fluxes. Thereby, we demonstrated that the previously root‐specific defined *BnaA02.NIP6;1a* isoform (Yuan *et al*., 2017) is actually highly expressed in the inflorescence (Figure 5b). Moreover, we demonstrated that BnaC05.NIP3;1b, BnaA05.NIP3;1b and NIP7 isoforms are strongly expressed in various inflorescence tissues, including the rachis and the open‐ and closed flowers. Diverse B‐permeable *NIP4s* were also detected in the inflorescence. In addition, several *NIP3* and *NIP4* genes were either significantly up‐ or downregulated dependent on the tissue and the plants’ B nutritional status, indicating that these channel‐types are actively regulated by this micronutrient and impact on its distribution. Especially our observations that BnaNIP4s are: (i) permeable to B; and (ii) expressed dependent on the plants’ B status are interesting with respect to the fact that in Arabidopsis, AtNIP4;1 and AtNIP4;2 seem to be required for pollen development and pollination, two processes that are highly dependent on sufficient B supply also in rapeseed (Di Giorgio *et al*., 2016). Whether the importance of Arabidopsis NIP4s in pollen development and pollination is due to their B transport function remains unknown to date.

In contrast to the root‐specific expression of *AtNIP5;1*, but in agreement with expression data from *B. napus* cvs. *Q10* and *W10* (Yuan *et al*., 2017), we detected *NIP5* transcripts also in shoot tissues and, so far not detected, in floral tissues. Four out of six *Darmor‐PBY018 NIP5s* were strongly upregulated in roots and shoots under B‐deficient conditions. *BnaA02.NIP5;1a* and *BnaC02.NIP5;1a* were upregulated in closed flower buds under B‐deficient conditions. No upregulation under limited B supply was observed for *BnaA07.NIP5;1c* and *BnaC06.NIP5;1c* under all tested condition (Figure 6b). Despite their high sequence similarity (Figure 1), this pair displays a completely differential expression pattern (Figure 5b). Together our results suggested that NIP5s are key transport regulators under B‐limiting conditions and ensure B fluxes throughout the plant body. *BnaA03.NIP5;1b*, which significantly contributes to B‐efficiency in cv.* Q10* (Hua *et al*., 2016a), was the B transporter with the highest total upregulation under B‐deficient conditions, namely 57‐fold in roots. Moreover, compared with the expression of other *NIP5s*,* BnaA03.NIP5;1b* was almost exclusively detected in roots. The strong B‐deficiency‐responsiveness of *BnaNIP5s* in roots and shoots was confirmed in Arabidopsis plants expressing three different *BnaNIP5*_*pro*_ *:GUS* constructs. For instance, the promoter activity of *BnaA03.NIP5;1b* but also of *BnaC02.NIP5;1a*, which were both found in B‐efficiency QTLs in cv. *Q10* (Hua *et al*., 2016a), were strongly enhanced under our B‐limiting conditions.

This specific regulation of *BnaNIP5* expression under B‐deficient conditions goes well in line with the existence of a minimum open reading frame (ORF), ATG‐Stop, in all six *BnaNIP5* promoters (Figure S2). It has been shown that this minimum ORF is crucial for controlling *AtNIP5;1* mRNA levels in dependence of the B supply conditions in Arabidopsis (Tanaka *et al*., 2016), and we hypothesize that the minimum ORFs of BnaNIP5 promoters have a similar conserved function in *B. napus*. Our qPCR data, obtained for *BnaA07.NIP5;1c* and *BnaC06.NIP5;1c*, demonstrated that further tissue‐ and developmental transcriptional control mechanisms must exist to regulate the activity of these two promoters in addition to the ATG‐Stop motif.

Together, with the provided evidence that these isoforms are highly permeable to B (Figure 2), these observations underlined the importance of these isoforms for B uptake transport processes in rapeseed. Interestingly, while we did not detect a significant upregulation of *BnaA07.NIP5;1c* by qPCR under B‐deficient conditions, we observed an obvious induced promoter activity in the GUS assays. This indicates that *BnaA07.NIP5;1c* transcript abundance is additionally developmentally regulated. *NIP5* promoter activity was strongly enhanced in the vasculature of root tips and in zones were lateral roots emerged. Whether BnaNIP5 isoforms play a role in lateral root emergence or whether this promoter activity is due to the fact that the activity was tested in a heterologous plant expression system will be further investigated. Moreover, in contrast to the *AtNIP5;1*_*pro*_, the *BnaNIP5;1* promoters were not active in the root epidermis of Arabidopsis.

The results of the GUS staining time series (Figures 7 and S3) indicate that the detected *BnaNIP5;1* promoter signals are not caused by a methodological artifact, but indeed represent the *BnaNIP5;1* promoter activities in the heterologous expression host Arabidopsis.

With this study, we provide comprehensive experimental evidence for B transport functions of six BOR1‐ and 13 NIP‐type *B. napus* B transporters exploiting transport assays in yeast (BOR1s) and oocytes (NIP2, NIP3, NIP4, NIP5, NIP6 and NIP7), as well as B transport complementation assays in Arabidopsis (NIP4, NIP5, NIP6 and NIP7). In combination with the generated tissue‐specific, developmental‐ and B‐status‐dependent expression maps for *BOR* and *NIP* genes, these results suggest that in particular NIP5s are key players in the routing of B fluxes throughout the plant. Additionally, we uncovered that members of other NIP subfamilies, such as NIP2, NIP3 and NIP4s, which have not yet been associated with B transport processes, have an impact on B transport regulation. Many BOR1s and NIPs are highly expressed within diverse tissues of the inflorescence or some are even flower‐specific, such as BnaC05.NIP7;1a, BnaA05.NIP7;1a and BnaA04.NIP4;1b. This indicates that the delivery of B to and its allocation within the flowers is tightly managed by both NIPs and BORs, and further highlights the role of B for the fertility of plants. Comparing our *Darmor‐PBY018* expression results with previous studies on *QY10* or *W10* (Yuan *et al*., 2017; Chen *et al*., 2018), we can conclude that cultivar‐specific B transporter expression characteristics exist. Such a diversity is fundamental for breeding strategies aiming at improving the B efficiency of Brassica crops to face future agricultural challenges.

## Experimental procedures

### Data resource, alignment and phylogenetic analysis of BORs and NIPs

Multiple public‐accessible genome databases were used for sequence retrievals. Bayesian phylogenetic analyses and tree computation were performed with curated protein alignments. Detailed information on these procedures is provided in Data S1.

### Cloning and vector construction

Information about vector constructions, used primers and the procedures for molecular cloning techniques is provided in Data S1.

### Oocyte transport assays

*In vitro* cRNA synthesis, oocyte handling procedures and various oocyte uptake assays with subsequent determination of permeability coefficients or HR‐ICP‐MS analysis‐based determination of element levels of oocytes are described in detail in Data S1.

### Complementation analysis of *Atnip5;1* T‐DNA insertion mutants

Detailed information on the T‐DNA insertion lines, vector constructions, the procedures for transgenic Arabidopsis generation and selection as well as the growth assay is provided in Data S1.

### Arabidopsis and *Brassica napus* growth experiments

Detailed information on growth conditions and treatments of Arabidopsis and *B. napus* cv. *Darmor‐PBY018* (Schmutzer *et al*., 2015) is given in Data S1.

### RNA extraction, cDNA synthesis and real‐time quantitative polymerase chain reaction

Various experimental information related to the different working steps necessary for a qPCR as well as qPCR‐related specification details are described in detail in Data S1.

### Yeast strains and growth assays

Detailed information on the yeast mutant lines *∆bor1* and *∆atr1* and the toxicity growth assay conditions are described in detail in Data S1.

### Data statement

The author responsible for distribution of materials integral to the findings presented in this article in accordance with the policy described in the Author Guidelines is: Gerd Patrick Bienert (bienert@ipk-gatersleben.de).

## Conflict of interest

The authors declare no conflicts of interest.

## Author contributions

Conceptualization: TAD and GPB; research: TAD (transport experiments in oocytes, promoter cloning), JF (qPCR, element analyses, B deficiency experiments with hydroponically grown B. napus plants), MDB (Arabidopsis growth complementation assays, element analyses, yeast growth assays, B deficiency experiments with soilgrown B. napus plants), AB (Arabidopsis and B. napus growth experiments on various B supply conditions), BP (qPCR, Arabidopsis transformation), ZL (GUS assays, B deficiency experiments with soil‐grown Arabidopsis plants), CS (GUS assays, B deficiency experiments with soil‐grown B. napus plants), NB (phylogenetic analyses), NR (transport experiments in oocytes), GPB (yeast growth assays); data analyses: TAD, JF, MDB, BP, AB, NB, ZL, NR, FC, GPB; writing – original draft: GPB; writing – review and editing: GPB. with the help of all authors.

## Supporting information


**Figure S1**. Tissue boron concentrations of *Brassica napus* cv.* PBY018* plants grown under B‐sufficient or B‐deficient growth conditions at the reproductive stage. Related to Figures 5 and 6.

**Figure S2**. Minimum ORFs (ATG‐Stop) upstream of *Brassica napus NIP5;1* genes.

**Figure S3.** *BnaNIP5;1* promoters are upregulated in Arabidopsis roots and shoots under B limitation.

**Data S1.** Supporting experimental procedures.
