# Supplementary figures and images for: Boron demanding tissues of Brassica napus express specific sets of functional Nodulin26‐like Intrinsic Proteins and BOR1 transporters
Source: Plant J. 2019 Jul 15;100(1):68–82. doi: 10.1111/tpj.14428 (PMC6852077; doi:10.1111/tpj.14428)

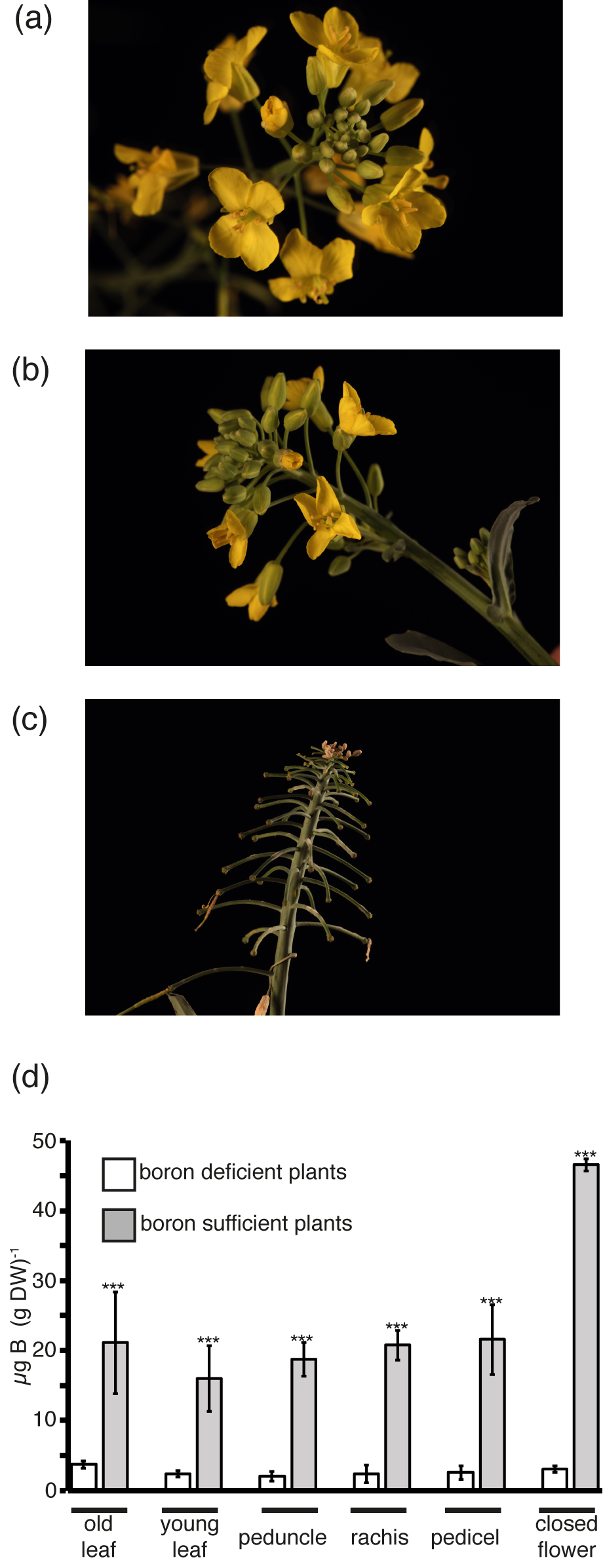

Supplement: Supplementary file 1 — Figure S1. Tissue boron concentrations of Brassica napus cv. PBY018 plants grown under B‐sufficient or B‐deficient growth conditions at the reproductive stage. Related to Figures 5 and 6. [file TPJ-100-68-s001.tif]

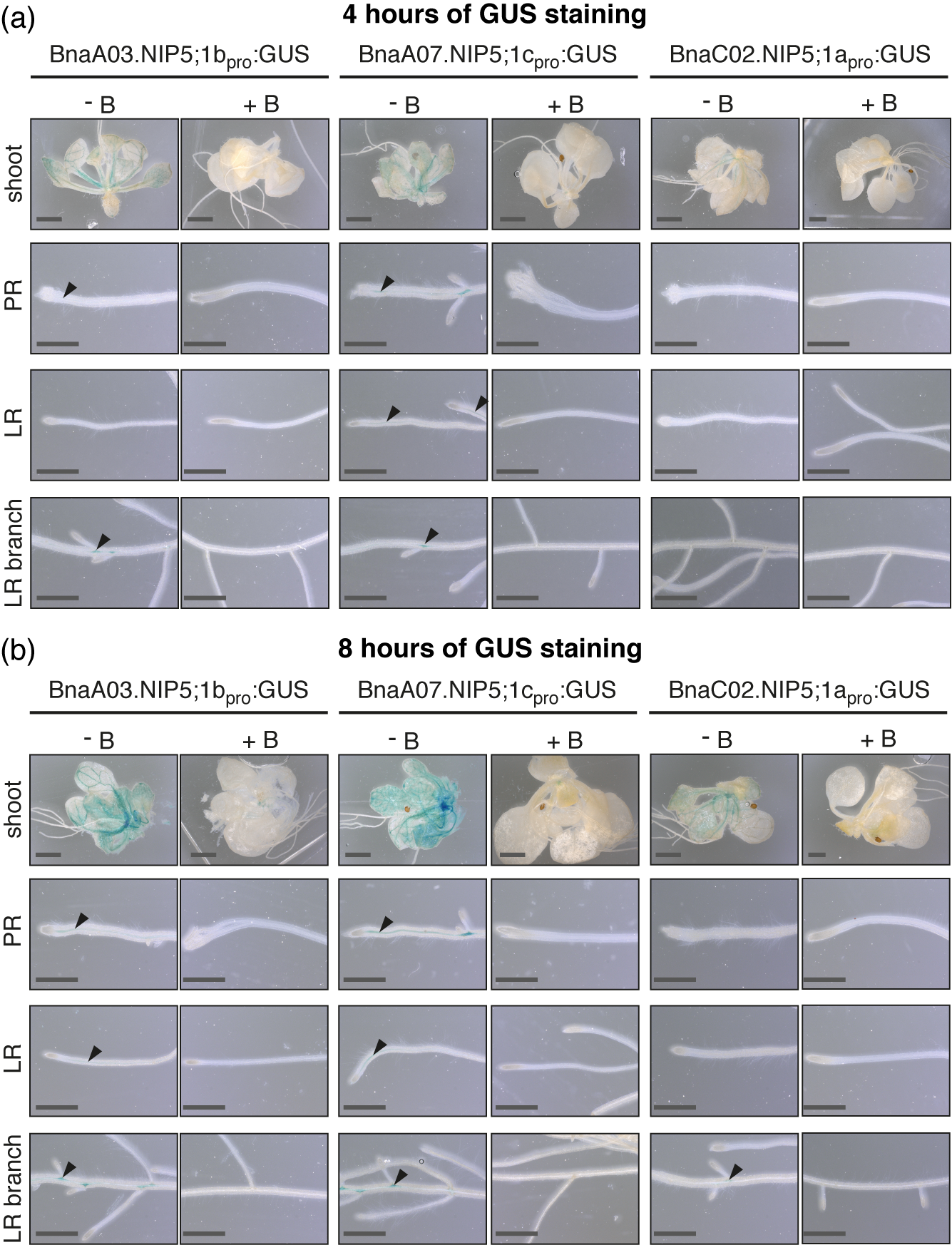

Supplement: Supplementary file 3 — Figure S3. BnaNIP5;1 promoters are upregulated in Arabidopsis roots and shoots under B limitation. [file TPJ-100-68-s003.tif]
