# Supplementary material for: Boron demanding tissues of Brassica napus express specific sets of functional Nodulin26‐like Intrinsic Proteins and BOR1 transporters
Source: Plant J. 2019 Jul 15;100(1):68–82. doi: 10.1111/tpj.14428 (PMC6852077; doi:10.1111/tpj.14428)
Supplement: Supplementary file 2 — Figure S2. Minimum ORFs (ATG‐Stop) upstream of Brassica napus NIP5;1 genes. [file TPJ-100-68-s002.docx]

(a)

BnaA02.NIP5_1a TG------------------------------------------------

BnaA03.NIP5_1b TTTTTG------------------------------------------AG

BnaA07.NIP5_1c TGGCT---------------------------------------------

BnaC02.NIP5_1a TC------------------------------------------------

BnaC03.NIP5_1b TTTTTG------------------------------------------AG

BnaC06.NIP5_1c TTGCCTTTGTTTTTTATAAGACCCGGTTTTTGACTTTGGTTTTGTGAGAG

*

BnaA02.NIP5_1a ----------------ATATTCCTCTTCTAACGTAGA----AACAGCTTT

BnaA03.NIP5_1b GTGGCTATCTTCGTTCATATTCTTCTTCTTACGTAGT----AACAGCTTC

BnaA07.NIP5_1c --ATCT-TCTTCGTTCATATTCTTCTTCTTACGTAGTAATTAACAGCTTT

BnaC02.NIP5_1a ----------------------------TAACGTAGA----AACAACTTT

BnaC03.NIP5_1b GTGGCTATCTTCGTTCATATTCTTCTTCTTACGTAGT----AACAGCTTT

BnaC06.NIP5_1c GTGGCTATCTTCGTTCATATTCTTCTTCTTACGTAGTAATTAACAGCTTT

* ****** **** ***

BnaA02.NIP5_1a ATAA-AATCCTTGAAAGCATGTAATTTAAAGTCTCAGATCCATTTTTTTA

BnaA03.NIP5_1b ATAAAAATCCTTACAAGCATGTAAATTAACGTCCCAGATCCATTTTCTCC

BnaA07.NIP5_1c ATAAAAATCCTTACAAGCATGTAAATTAACGTCCCAGATCCATTTTCTTC

BnaC02.NIP5_1a ATAA-AATCCTTCAAAGCATGTAATTTAAAGTCTCAGATCCATTTTCTTC

BnaC03.NIP5_1b ATAAAAATCCTTACAAGCATGTAAATTAACGTCCCAGATCCATTTTCTTC

BnaC06.NIP5_1c ATAAAAATCCTTACAAGCATTTAAATTAACGTCCCAGATCCA--------

**** ******* ****** *** **** *** ********

BnaA02.NIP5_1a TACTTGCAAAAAAAA--AAAAAACT----TATAGATAAAA-TTTTCATAA

BnaA03.NIP5_1b TCCTTCAAAAGAAAAAGAAAATTTTAATCGGTAGATA--A-GTTTGATTC

BnaA07.NIP5_1c TCCTTAATTATAAAA--AAGATTTTAATCGATAGATA--AGTTTTCATTC

BnaC02.NIP5_1a TACTTGCAAAAAAAA--AAAAAATTAACTTATATATAAAA-TTTTCATAA

BnaC03.NIP5_1b TCCTTCAGAAAAAAA--AAAATTTTAATCGGTAGATA--A-GTTTGATTC

BnaC06.NIP5_1c -----------------------------------------TTTTCATTC

*** **

BnaA02.NIP5_1a ATTGGTGTCTCTCTCGTTTTAATTTGTGTTTGGTGAAACAAGTCCTGGCA

BnaA03.NIP5_1b ATTGGCGTC--CCTGGTTTTAATTTCTGTTTGGTGAAACTAGTCCTGGCA

BnaA07.NIP5_1c ATTTGTGTC--TCTGGTTTTAATTTGTGTTTGGTGAAACTAGTACTGGCA

BnaC02.NIP5_1a ATTGGTGTCTCTCTCGTTTTAATTTGTGTTTGGTGAAACAAGTCCTGGCA

BnaC03.NIP5_1b ATTGGTGTC--CCTGGTTTTAATTTGTGTTTGGTGAAACTAGTCCTGGCA

BnaC06.NIP5_1c ATTTGTGAC--TCTGGTTTTAATTTGTGTTTGGTGAAACTAGTACTGGCA

*** * * * ** ********** ************* *** ******

BnaA02.NIP5_1a AAGTTTAAAAC-ACAAGTGTAAACCCAAATTTATAAAAAACTTCAAATC-

BnaA03.NIP5_1b AAGTTTGAAGC-ACAAGTGTAAACCCAAATTAAAAA-AAACCTCAAATC-

BnaA07.NIP5_1c AAACTTGAAGCAACAAGTGTAAACCCAAATTTATA---AACGTCAAATC-

BnaC02.NIP5_1a AAGTTTAAAAC-ACAAGTGTAAACCCAAATTTATAA-AAACTTCAAATC-

BnaC03.NIP5_1b AAGTTTGAAGC-ACAAGTGTAAACCCAAATTTATAA-AAACCTCAAATC-

BnaC06.NIP5_1c AAACTTGAAGC-ACAAGTG--AACCCAAATTTATA---AACGTCAAATC-

** ** ** * ******* ********** * * *** ********

BnaA02.NIP5_1a **ATGTAA**ATTTCGTTTCTATTCAATTTATCTACCTTCC-CCC--AAAAAA--

BnaA03.NIP5_1b **ATGTAA**ATTTCGTCTCT-TCAAATTTATCTCCCT-CC-TAC--AAAAAA--

BnaA07.NIP5_1c **ATGTAA**AATTCGTCTCTATTAAATTTATCTTCCT-CC-TAC--AAAAAA--

BnaC02.NIP5_1a **ATGTAA**ATTTCGTTTCTATTCAATTTATCTACCTTCCCCCCAAAAAAAAAA

BnaC03.NIP5_1b **ATGTAA**ATTTCGTCTCT-CGAAATTTATCTCCCT-CC-TAC--AAAAAA--

BnaC06.NIP5_1c **ATGTAA**AATTCGTCTCTATTAAATTTATCTCCCT-CC-TAC--AAAAAA--

******* ***** *** ********* *** ** * ******

BnaA02.NIP5_1a -ACAAAAAAAAT--CAGAATTTAGAATTTTTATTTAAAACTAAAAAAGAA

BnaA03.NIP5_1b -ACAAAAAAAA----AGA----AGTATT-TTCTTCAAAAC-AAAATACAA

BnaA07.NIP5_1c -AAAACAAAA-----AAA----AGTATT-TTCTTCAAAAT-AAAATAAAT

BnaC02.NIP5_1a AACAAAAAAAAAATCAGAATTTAGGATTTTTATTTAAAACTAAAAAAGAA

BnaC03.NIP5_1b -AGAAAAAAAA----AGA----AGTATT-TTCTTCAAAAC-AAAATACAA

BnaC06.NIP5_1c -ACCAAAAAA-----AAA----AGTATT-TTCTTCAAAAT-AAAATACAA

* * **** * * ** *** ** ** **** **** * *

BnaA02.NIP5_1a ACAAAAAGCCCCAA-AA-AAAGAGTGAGAGTTTGGAA

BnaA03.NIP5_1b AA--AA--TACCCG-A--AAAG--------ATT-AGG

BnaA07.NIP5_1c AAAAAAACTACCCG-A--AAAG--------GTT-AGG

BnaC02.NIP5_1a ACAAAAAGCCCAAAAAAAAAAGAGTGAGAATTT-GGA

BnaC03.NIP5_1b AAAGAA--TACCAG-A--AAAG--------GTT-AGG

BnaC06.NIP5_1c AAAAAAACTACCCG-A--AAAG--------GTT-AGT

* ** * * **** **

(b)

| NIP5 gene name | ATG-Stop sequence | ATG-Stop position relative to the corresponding NIP5 start codon |
| --- | --- | --- |
| BnaC06.NIP5;1c | ATGTAA | -103 to -108 |
| BnaA02.NIP5;1a | ATGTAA | -123 to -128 |
| BnaC03.NIP5;1b | ATGTAA | -101 to -106 |
| BnaC02.NIP5;1a | ATGTAA | -132 to -137 |
| BnaA07.NIP5;1c | ATGTAA | -103 to -108 |
| BnaA03.NIP5;1b | ATGTAA | -099 to -104 |
