# Supplementary material for: Boron demanding tissues of Brassica napus express specific sets of functional Nodulin26‐like Intrinsic Proteins and BOR1 transporters
Source: Plant J. 2019 Jul 15;100(1):68–82. doi: 10.1111/tpj.14428 (PMC6852077; doi:10.1111/tpj.14428)
Supplement: Supplementary file 4 — Data S1. Supporting experimental procedures. [file TPJ-100-68-s004.docx]

**Supplemental Experimental Procedures**

**Data Resource, alignment and phylogenetic analysis of BORs and NIPs**

*Arabidopsis thaliana*, *Brassica napus cvs. Darmor-bzh* and Z*hongshuang11* genomic and annotation data were downloaded from the TAIR10 (http://www.arabidopsis.org/index.jsp), the CNS-Genoscope (http://www.genoscope.cns.fr/ brassicanapus/) and the Genebank genome BLAST search function (https://www.ncbi.nlm.nih.gov/genome/203?genome_assembly_id=59327) databases, respectively. The *B. napus* sequences were checked manually in comparison with known sequences for correctness of annotation, and intron–exon borders. Genomic, cDNA as well as protein sequences were corrected when necessary.

A multiple amino acid sequence alignment for both, the BOR and the NIP data set, was generated using MAFFT v7.222 (Katoh et al., 2002) and the BLOSUM matrix 45 (Heniko and Heniko, 1992) as implemented in GENEIOUS PRO v10.2.3 (Kearse et al., 2012). The alignments were checked by eye and non-homologous regions were trimmed. A distance matrix of the final alignment was used to ensure that proteins are still homologous and suitable for phylogenetic analysis i.e. that they differ by less than 80-85% (Opperdoes, 2003). Bayesian inference (BI) was performed in MRBAYES 3.2.6 (Ronquist and Huelsenbeck, 2003). Both analyses were run with two independent Metropolis coupled MCMC analyses each with four sequentially heated chains (temperature set to 0.05), running for three million generations and tree sampling every 500 generations. We allowed for model jumping between amino acid substitution models by setting ‘prset aamodelpr = mixed’. The continuous parameter values sampled from the chains were checked for mixing using TRACER v1.6 (Rambaut and Drummond, 2007). A consensus tree was computed after deleting a burn-in of the first 25% of trees and visualized in FIGTREE v1.4.2 (Rambaut A. FIGTREE v1.4.2. Available from: http://tree.bio.ed.ac.uk/software/figtree. accessed 20 Mar 2015) using midpoint rooting.

**Cloning and vector construction**

Primers matching *NIP* (Nr.1 to Nr.28) and *BOR*1 (Nr.45 to Nr.58) sequences were used to PCR amplify *NIP* and *BOR*1 cDNA sequences and generate the respective constructs (Supplemental Experimental Procedures: Cloning-Primer-Table): PCR products were cloned into the USER-compatible *X. laevis* expression vector pNB1u (NIPs) or the *Saccharomyces cerevisiae* expression vector pYeDP60u (BORs) using an uracil excision-based improved high-throughput USER cloning technique (Nour-Eldin et al., 2006). Primers (Nr.29 to Nr.34; Supplemental Experimental Procedures: Cloning-Primer-Table) matching three *BnaNIP5* promoter sequences were used to PCR amplify the *BnaNIP5* promoter fragments (Supplemental Experimental Procedures: Promoter-Table). PCR products were USER-cloned into the compatible plant expression vector pCambia3300_NLS_GFP_GUSu containing a GFP-GUS fusion reporter gene in which the GFP is targeted to the nucleus by a nuclear localization signal (NLS) yielding the expression clones PL103 (*BnaC02.NIP5;1a_pro_:GUS*), PL105 (*BnaA07.NIP5;1c_pro_:GUS*), and PL106 (*BnaA03.NIP5;1b_pro_:GUS*).

*AtNIP5;1*, *LOC106388529* (NIP5;1), *BnaC06.NIP6;1a*, *BnaC05.NIP7;1a*, *BnaC04.NIP4;1b*, and *BnaC04.NIP4;1a* cDNAs were amplified using the primers (Nr.35 to Nr.44) listed in Supplemental Experimental Procedures: Cloning-Primer-Table. Resulting PCR fragments were digested with XhoI and AscI, purified and ligated behind the *AtNIP5;1* promoter fragment into pMT15 cut with XhoI and AscI. Resulting vectors (pMT17 (*BnaC04.NIP4;1b*), pMT18 (*BnaC04.NIP4;1a*), pMT23 (*LOC106388529, NIP5*), pMT25 (*BnaC06.NIP6;1a*) and pMT27 (*BnaC05.NIP7;1a*)) were subjected to LR reactions using the Gateway LR Clonase Plus II Enzyme Mix (Life Technologies, USA) together with the destination vector pGWB1 (Nakagawa et al., 2007) yielding the expression clones PL51 (*AtNIP5;1*), PL95 (*LOC106388529, NIP5*), PL97 (*BnaC06.NIP6;1a*), PL98 (*BnaC05.NIP7;1a*), PL99 (*BnaC04.NIP4;1b*), and PL100 (*BnaC04.NIP4;1a*).

All sequences were verified by sequencing.

***In vitro* RNA synthesis**

Ready-to-use capped complementary RNAs encoding MIP sequences were synthesized *in vitro* using the mMESSAGE mMACHINE T7 Transcription Kit (Thermo Fisher Scientific, Germany). Oocytes were isolated, from adult female *X. laevis* frogs, and placed in a Barth's buffer solution. Oocytes were treated with 0.1% collagenase type B (Roche Diagnostics) in a calcium-free Barth buffer for 1.5 h to remove follicular cell layers, washed five times with collagenase-free Barth buffer, and selected according to the size and development stage. Selected oocytes were incubated for one day in Barth Buffer at 20 °C prior to cRNA injection.

**Oocyte transport assays**

For the water transport assay, oocytes were injected with 50 nL of the different aquaporin cRNAs (12.5 ng *NIP* or 2.5 ng *PIP* cRNA per oocyte) or an equal volume of RNase-free water as a negative control. Oocytes were kept at 20 °C for 2-3 d in Barth buffer prior to experiments. The P_f_ (osmotic water permeability coefficient) was determined as described previously (Fetter et al., 2004).

For the boric acid and arsenous acid uptake assays, oocytes were injected with 50 nL of diverse aquaporin cRNAs (12.5 ng per oocyte) or equal volume of RNase-free water as a negative control. After three days, oocytes (8-12 oocytes per replicate; 5-10 replicates) were incubated for distinct time points (B: 0 and 20 min; As: 0, 15, and 30 min) in Barth buffer (pH 7.4) containing 5 mM ^10^Boric acid (Sigma-Aldrich) or 0.1 mM µM NaAsO_2_ (Sigma Aldrich). Afterwards, the oocytes were washed three times in Barth buffer with 5 mM ^11^Boric acid (Sigma Aldrich) or without NaAsO_2_, respectively, and dried before the elemental analysis was performed. Uptake assays were repeated with oocytes isolated from two or three different frogs resulting in consistent results.

**HR-ICP-MS analysis**

For elemental analysis, oocytes and plant material were dried at 65°C. Approximately ten milligram of plant dry matter or the oocytes were digested in nitric acid (HNO_3_) using a high-performance microwave reactor (UltraClave IV; MLS GmbH, Leutkirch, Germany). The elemental analysis was performed with a sector field high-resolution ICP-MS (Element 2, Thermo Fisher Scientific, Waltham, MA, USA) using software v.3.1.2.242 (Eggert and von Wirén, 2013).

**Plant material and cultivation**

*A. thaliana* wild type (Col-0) seeds or the T-DNA insertion line for AtNIP5;1 (SALK_122287), described by Takano *et al.* (Takano et al., 2006) has been obtained from NASC (N622287), were surface sterilized with 70% ethanol plus 0.05% Triton X100 followed by three washes with 99% ethanol. Dry seeds were sown on half-strength MS (Murashige and Skoog, 1962) medium (2.2 g/L MS salts with minimal organics (Sigma), 1% sucrose, 0.7% agar, pH 5.8 (KOH)) with or without antibiotic. Seeds were vernalized for 2 days at 4 °C. In vitro cultures were grown in a photoperiod of 10 h light at 22 °C (120 μmol m^−2^ sec^−1^) and 14 h dark at 19 °C. After germination and seedling development, plants were transferred to either soil substrate or sterile culture medium.

*Brassica napus cv. Darmor-*PBY018 (Schmutzer et al., 2015) were cultivated in either B-deficient or B-sufficient soil substrate in the green house, in B-deficient or B-sufficient hydroponic culture or in near-field conditions as described previously (Pommerrenig et al., 2018).

Greenhouse cultivation: In short, B-free zerosoil-substrate was supplemented with 0.5% CaCO_3_ and 0.3% CaO. Calcium concentration was adjusted by mixing 20 kg soil with 1 L of CaCO_3_ (100 g L^-1^) and 1 L of CaO (60 g L^-1^) in a clean cement mixer for 10 min. Thereafter, soil substrate was manually mixed each other day. About 1-2 weeks after preparation clumps were sieved out and the soil (dry matter: ~ 30 %) was used for the experiments*.* Rapeseed plants germinated and have been grown in pots filled with zerosoil-substrate in the greenhouse. Plants were watered per pot every two days with 250 ml 1x Hoagland solution containing 200 µM boric acid. All solutions additionally contained the following concentrations of nutrients: 7.5 mM NH_4_NO_3_, 2.9 mM KH_2_PO_4_, 850 µM MgSO_4_, 340 µM K_2_SO_4_, 320 µM MnCl_2_, 52 µM CuSO_4_, 20 µM NaFeEDTA, 4.5 µM ZnSO_4_, 0.4 µM NaMoO_4_.

At BBCH15, and prior to the vernalization (6 weeks at 6°C), plants were re-planted into new pots under the same watering and fertilization management condition but without the addition of any B to the soil substrate. Care was taken that little soil was sticking to the roots before they were replanted. After the vernalization, plants were transferred to the greenhouse. No glassware was used in any process during the preparation of the nutrient solution or the irrigation of the plants to prevent additional input of B into the growth system. Nutrients were supplied to the soil weekly with the above-described nutrient solution without B. Greenhouse conditions were set to long-day conditions (16h day/8h night) at 22/18 °C, 60/70% relative humidity, and 250  µmoles photons PAR m^-2^ s^-1^ light intensity. At BBCH61, plant tissues were harvested for RNA isolation and elemental analysis.

Hydroponic cultivation: Seed were sterilized with 4.2% NaClO for 10 min at 37 °C, washed 3x with sterile milliQ H_2_O and dried on a filter paper. Seeds were placed on 0.5x Hoagland (0.3% agar-agar, Ca(NO_3_)_2_ (2.5 mM), MgSO_4_ (2.5 mM), KNO_3_ (2.5 mM), KH_2_PO_4_ (0.5 mM), FeNaEDTA (5 µM), MnCl_2_ (2.25 µM), ZnSO_4_ (1.9 µM), CuSO_4_ (0.15 µM), (NH_4_)_6_Mo_7_O_24_ (0.05 µM), and MES (pH 5.8, 1 mM), pH 5.8 (KOH)), stratified for 2 days at 4 °C and transferred to a climate chamber (300 - 320 µmol light; 16h day (22 °C); 8h night (20 °C); 60% humidity) for germination. Three days after germination (DAG), hypocotyls of seedlings were surrounded by foamed material and transferred to a 2 cm lid-opening of brown 50 ml Falcon-tubes filled with 0.5x Hoagland either containing 0 µM (B-deficient) or 25 µM B (B-sufficient). Medium was refilled each day and a complete exchange was performed each three days. 13 DAG, plants were transferred to a 2 cm lid-opening of brown 1 L closed plastic bottles filled with 0.5x Hoagland solution either containing 0 µM (B-deficient) or 25 µM B (B-sufficient) and the nutrient solution was aerated. Medium was refilled each day and a complete exchange was performed at 17 DAG. 21 DAG, plant tissues (roots, leaves, stem and leaf stalks) were harvested for RNA extraction.

Near-field-greenhouse growth conditions: *Darmor-PBY018* plants were germinated on standard soil substrate, vernalized from BBCH 15 onwards for 6 weeks (6 °C) and transferred to a near-field-greenhouse at IPK Gatersleben. Plants were watered and fertilized according to their demand. At BBCH 61, plant tissues were harvested for RNA extraction.

**Generation of transgenic Arabidopsis lines**

Above-described PL expression constructs were introduced into *Agrobacterium tumefaciens* (LBA4404 virGN54D; van der Fits et al., 2000) for the subsequent transformation of the Arabidopsis *Atnip5;1* T-DNA insertion mutant (PL51, PL95, PL97, PL98, PL99, PL100 ) or Col-0 wild type plants (PL103, PL105 and PL106) using the floral dip method (Clough and Bent, 1998). Transgenic *Atnip5;*1 or Col-0 plants were selected on agar plates with MS-medium supplemented with 0.5% sucrose and 25 µg mL^-1^ hygromycin or 50 µg mL^-1^ kanamycin sulfate. Hygromycin- and kanamycin resistant plants from each line were transferred to standard IPK soil substrate and amplified.

**Complementation analysis of *Atnip5;1* T-DNA insertion mutants**

T2 plants were germinated on half-strength MS medium (2.2 g/L MS salts with minimal organics (Duchefa), 1% sucrose, 0.7% Phytagel (Sigma), pH 5.8 (KOH)) containing hygromycin. After one week, the plantlets were transferred to B deficiency medium (0.2 µM H_3_BO_3_, 625 µM KH_2_PO_4_, 750 µM MgSO_4_, 1.5 mM CaCl_2_, 9.4 mM KNO_3_, 1 mM NH_4_NO_3_, 75 µM FeNa_2_EDTA, 0.055 µM CoCl_2_, 0.05 µM CuSO_4_, 50 µM MnSO_4_, 0.5 µM Na_2_MoO_4_, 15 µM ZnSO_4_, 2.5 µM KI, 1 mM MES, pH 5.5 (KOH), 1% Phytagel). The B deficiency medium was treated with milliQ-washed B-chelator Amberlite IRA-743 (3 g/L, Sigma) overnight prior to autoclaving to avoid any contaminations of B. After 7 days of growth, roots were harvested for RNA extraction. The *Atnip5;1* mutant genotype was always confirmed for the individual transformants by PCR. Successful heterologous expression of *BnaNIPs* in the roots of *Atnip5;1* transformants was tested using the primers (Nr.59 to Nr.72) listed in Supplemental Experimental Procedures: RT-PCR-Primer-Table. *BnaNIP* expressing plants were used to generate the T3 generation which was characterized for their growth behavior on standard IPK greenhouse soil.

**RNA extraction, cDNA synthesis, and real-time quantitative PCR**

About 10-40 mg *B. napus* cv. *Darmor-PBY018* plant material was harvested from plant tissues, immediately frozen in liquid nitrogen and grounded by a mortar in liquid nitrogen. Total RNA was extracted using NucleoSpin RNA Plant Kits and DNase I treatment according to the manufacturer’s instructions (Macherey-Nagel, Germany). CDNA was synthesized out of 1000 ng total RNA using MuLV-Reverse Transcriptase (Fermentas, Germany) in a total volume of 20 µl and diluted to 1:20 with nuclease free water. RT qPCR was performed in a 384 well thermocycler (CFX384 TouchTM Real-Time PCR Detection System, Bio-Rad) using the GoTaq qPCR Mastermix (Promega, USA). Two µl diluted cDNA were used per reaction. Three identically treated biological replicates were run. The thermocycler protocol was as follows: 3 min at 95 °C, followed by 45 cycles of 10 s at 95 °C, and 50 s at 58 or 60 °C. Specificity of the primers was validated by BLASTN against the Genoscope and the NCBI database, by visualizing amplified PCR products on agarose gels and/or by sequencing of the amplified PCR products. For the generation of a standard curve, aliquots of above described cDNAs of diverse tissue pools of the corresponding growth conditions were used in a mixture. These mixtures were serially diluted (1 to 1, 1 to 2, 1 to 4, 1 to 8, 1 to 16, 1 to 32, and 1 to 64) with nuclease free water to generate standard curve templates and to determine PCR efficiencies for each primer pair. Assays producing PCR efficiencies > 80% < 115% were used for expression data analyses. Cq values > 40 were not used for quantification. Relative expression of each *NIP* or *BOR1* gene was determined by qPCR and calculated using a gene expression normalization factor (calculated by GeNorm software (Vandesompele et al., 2002)) for each tissue sample based on the geometric mean of three reference genes (Bna.EF, Bna.Exp and Bna.TIP4;1) exhibiting stability factors (M) below 1.5. To calculate the ∆Ct for each *NIP* or *BOR1* gene of interest, the tissue probe which resulted in the lowest Ct value was chosen as a reference for the respective B-transporter gene. Relative expression values are displayed in a heat map representation which was generated using the Morpheus software (https://software.broadinstitute.org/morpheus).

**Promoter-GUS Analysis**

BnaNIP5;1_pro_:GUS lines were germinated in half-strength MS medium (2.2 g/L MS salts with minimal organics (Duchefa), 1% sucrose, 0.7% Phytagel (Sigma), pH 5.8 (KOH)). After seven days, seedlings were transferred to B-deficient (0.1 µM B) or B-sufficient (100 µM B) medium (which additionally contained: 625 µM KH_2_PO_4_, 750 µM MgSO_4_, 1.5 mM CaCl_2_, 9.4 mM KNO_3_, 1 mM NH_4_NO_3_, 75 µM FeNa_2_EDTA, 0.055 µM CoCl_2_, 0.05 µM CuSO_4_, 50 µM MnSO_4_, 0.5 µM Na_2_MoO_4_, 15 µM ZnSO_4_, 2.5 µM KI, 1 mM MES, pH 5.5 (KOH), 1% Phytagel). After 9 days in the main culture plants were incubated at 37 °C in a freshly prepared GUS reaction buffer (50 mM phosphate buffer (pH 7.2), 2 mM potassium ferrocyanure, 2 mM potassium ferriccyanure and 2 mM 5-bromo-4-chloro-3-indolyl-β-d-glucuronide) for 4, 8, or 16 h and de-stained with 70% ethanol. Samples were imaged with a light microscope (Axioskop; Carl Zeiss).

**Yeast strains**

For the boric acid toxicity growth assay, the yeast strains *∆bor1*/YNL275w/Y01169/BY4741 and *∆atr1*/YML116w/Y06516/BY4741 (EUROSCARF) and indicated BOR1s of Arabidopsis and *B. napus* were used. For the toxicity growth assay, the different *S. cerevisiae* deletion mutant strains were transformed with either the empty vector pYeDP60u (negative control) or pYeDP60u containing the respective *BOR1* cDNAs. AtBOR1 was used as a positive control.

**Growth assays**

Transformed *∆bor1* and *∆atr1* yeast mutant cells were selected on synthetic medium containing 2% glucose, 50 mM succinic acid/Tris base, pH5.5, 0.7% yeast nitrogen base without amino acids (Difco) and 2% agar. For the boric acid toxicity growth assays, 2% galactose, 50 mM succinic acid/Tris base, pH 5.5, 0.7% yeast nitrogen base without amino acids (Difco) and different concentrations of boric acid were used for the growth media. All media were supplemented according to the auxotrophic requirements with histidine, leucine, and methionine. Yeast cells were diluted to different OD_600_ values (1, 0.01, and 0.0001). Growth was documented after 7-10 days at 30°C. Three independent growth experiments were performed with each yeast strain and all with consistent results.

**Cloning-Primer-Table: Primers which have been used in this study for cloning:**

| Primer | | Sequence | Destination Vector |
| --- | --- | --- | --- |
| Nr. | Name |  |  |
| 1 | FW_pOO130_AtNIP5;1 | GGCTTAA(2-Deoxyuridine)ATGGCTCCACCAGAAGCTGAAG | pNB1u |
| 2 | RV_pOO130_AtNIP5;1 | GGTTTAA(2-Deoxyuridine)TTATCTTCTGAAAGATCTAACTGGTC | pNB1u |
| 3 | FW_pOO135_BnaC08.NIP3;1c | GGCTTAA(2-Deoxyuridine)ATGGCTGAGATCTCTGATAC | pNB1u |
| 4 | RV_pOO135_BnaC08.NIP3;1c | GGTTTAA(2-Deoxyuridine)TTATGGCAACTTGCATGTGAC | pNB1u |
| 5 | FW_pOO155_BnaA05.NIP3;1b | GGCTTAA(2-Deoxyuridine)ATGGCCGAGATCTCTGGTATTAC | pNB1u |
| 6 | RV_pOO155_BnaA05.NIP3;1b | GGTTTAA(2-Deoxyuridine)TTATGCCAACTTGCAAGTGAC | pNB1u |
| 7 | FW_pOO116_pP176_BnaC04.NIP4;1a | GGCTTAA(2-Deoxyuridine)ATGACTTCGCATGTTGAAGAAATTG | pNB1u |
| 8 | RV_pOO116_pP176_BnaC04.NIP4;1a | GGTTTAA(2-Deoxyuridine)TTAGCTTTTAGAAGTAGAAGTTTTG | pNB1u |
| 9 | FW_pOO145_BnaA04.NIP4;1a | GGCTTAA(2-Deoxyuridine)ATGACTTCGCATGGTGAAGGCATC | pNB1u |
| 10 | RV_pOO145_BnaA04.NIP4;1a | GGTTTAA(2-Deoxyuridine)TTATCTGTTAGGACCAGGAGAAGCG | pNB1u |
| 11 | FW_pOO106_pP177_BnaA02.NIP5;1a | GGCTTAA(2-Deoxyuridine)ATGGCTCCAACGGAGGCTGAG | pNB1u |
| 12 | RV_pOO106_pP177_BnaA02.NIP5;1a | GGTTTAA(2-Deoxyuridine)TTAACGGCGGAAGCTCCTAACCTGAC | pNB1u |
| 13 | FW_pOO108_BnaC02.NIP5;1a | GGCTTAA(2-Deoxyuridine)ATGGGTCCAACGGAGGCTGAG | pNB1u |
| 14 | RV_pOO108_BnaC02.NIP5;1a | GGTTTAA(2-Deoxyuridine)TTAACGGCGGAAGCTCCTAACCTGAC | pNB1u |
| 15 | FW_pOO109_BnaA03.NIP5;1b | GGCTTAA(2-Deoxyuridine)ATGTCTCCGCCGGAGGCTGAAATG | pNB1u |
| 16 | RV_pOO109_BnaA03.NIP5;1b | GGTTTAA(2-Deoxyuridine)TTAACGACGGAAGCTTCTAACCTGACGTGGC | pNB1u |
| 17 | FW_pOO110_BnaC06.NIP5;1c | GGCTTAA(2-Deoxyuridine)ATGTCTCCACCAGAAGCTG | pNB1u |
| 18 | RV_pOO110_BnaC06.NIP5;1c | GGTTTAA(2-Deoxyuridine)TTATCTTCTGAAAGATCTAACTTGTC | pNB1u |
| 19 | FW_pOO111_BnaA02.NIP6;1a | GGCTTAA(2-Deoxyuridine)ATGGACCACGAGGAGATTCC | pNB1u |
| 20 | RV_pOO111_ BnaA02.NIP6;1a | GGTTTAA(2-Deoxyuridine)TCATCTCCTGAAGCTCCGCTTCTC | pNB1u |
| 21 | FW_pOO112_pP183_BnaC06.NIP6;1a | GGCTTAA(2-Deoxyuridine)ATGGATCACGAGGAAATACC | pNB1u |
| 22 | RV_pOO112_pP183_BnaC06.NIP6;1a | GGTTTAA(2-Deoxyuridine)TCATCTCCTGAAGCTCCGCTTCTC | pNB1u |
| 23 | FW_pOO136_BnaA05.NIP2;1a | GGCTTAA(2-Deoxyuridine)ATGGATGACATCTCAGTGAGC | pNB1u |
| 24 | RV_pOO136_BnaA05.NIP2;1a | GGTTTAA(2-Deoxyuridine)TTACAAAGGAAGATCAGTAATTC | pNB1u |
| 25 | FW_pOO148_BnaA07.NIP6;1b | GGCTTAA(2-Deoxyuridine)ATGGACCACGAGGAGATTCC | pNB1u |
| 26 | RV_pOO148_BnaA07.NIP6;1b | GGTTTAA(2-Deoxyuridine)TCATCTTCTGAAGCTCCTCTTTTC | pNB1u |
| 27 | FW_pOO114_pP185_BnaA05.NIP7;1a | GGCTTAA(2-Deoxyuridine)ATGAATGTTGAGGTACGGTC | pNB1u |
| 28 | RV_pOO114_pP185_BnaA05.NIP7;1a | GGTTTAA(2-Deoxyuridine)TTAGCGTAACAGGGAAGAGACC | pNB1u |
| 29 | FW_PL103_pBnaC02.NIP5;1a | GGCTTAA(2-Deoxyuridine)CCTCAAGTGGTGTTTCGGTC | pCambia 3300 NLS-GFP-GUSu |
| 30 | FW_PL105_pBnaA07.NIP5;1c | GGCTTAA(2-Deoxyuridine)TCACAAGCGTTTAACTGCGT | pCambia 3300 NLS-GFP-GUSu |
| 31 | FW_PL106_pBnaA03.NIP5;1b | GGCTTAA(2-Deoxyuridine)GAAGGTTTTGTGAGGCCCAG | pCambia 3300 NLS-GFP-GUSu |
| 32 | RV_PL103_BnaC02.NIP5;1a | GGTTTAA(2-Deoxyuridine)TCCAAATTCTCACTCTTTTTTTTTTG | pCambia 3300 NLS-GFP-GUSu |
| 33 | RV_PL105_BnaA07.NIP5;1c | GGTTTAA(2-Deoxyuridine)CCTAACCTTTTCGGGTAG | pCambia 3300 NLS-GFP-GUSu |
| 34 | RV_PL106_BnaA03.NIP5;1b | GGTTTAA(2-Deoxyuridine)CCTAATCTTTTCGGGTATTTTTTG | pCambia 3300 NLS-GFP-GUSu |
| 35 | FW_PL95_LOC106388529 | gagagactcgagATGTCTCCGCCAGAGGCTGAAATG | pGWB1 |
| 36 | RV_PL95_LOC106388529 | ctctctggcgcgccTTAACGACGGAAGCTTCTAACCTGACGCGGTG | pGWB1 |
| 37 | FW_PL97_BnaC06.NIP6;1a | gagagactcgagATGGACCACGAGGAGATTCC | pGWB1 |
| 38 | RV_PL97_BnaC06.NIP6;1a | ctctctggcgcgccTCATCTCCTGAAGCTCCGCTTCTC | pGWB1 |
| 39 | FW_PL98_BnaC05.NIP7;1a | gagagactcgagATGAATGTTGAGGTACGGT | pGWB1 |
| 40 | RV_PL98_BnaC05.NIP7;1a | ctctctggcgcgccTTAGCGTAACAGGGAAGAG | pGWB1 |
| 41 | FW_PL99_BnaC04.NIP4;1b | gagagactcgagATGACTTCTCACGGTGAAGG | pGWB1 |
| 42 | RV_PL99_BnaC04.NIP4;1b | ctctctggcgcgccTTAGCTGTTAGAACTAGAAGCATTG | pGWB1 |
| 43 | FW_PL100_BnaC04.NIP4;1a | gagagactcgagATGACTTCGCATGTTGAAGAAATTG | pGWB1 |
| 44 | RV_PL100_BnaC04.NIP4;1a | ctctctggcgcgccTTAGCTTTTAGAAGTAGAAGTTTTG | pGWB1 |
| 45 | FW_pSc449_AtBOR1 | GGATTAA(2-Deoxyuridine)AATGGAAGAGACTTTTGTGCCG | pYeDP60u |
| 46 | RV_pSc449_AtBOR1 | GGGTTAA(2-Deoxyuridine)TCAGTTCGATGACGACTGGTTC | pYeDP60u |
| 47 | FW_pSc482_BnaC03.BOR1;3c | GGATTAA(2-Deoxyuridine)AATGGAAGAGACTTTCGTGCCG | pYeDP60u |
| 48 | RV_pSc482_BnaC03.BOR1;3c | GGGTTAA(2-Deoxyuridine)TCAATGAGAGCAATGGTTCAAAG | pYeDP60u |
| 49 | FW_pSc483_BnaA03.BOR1;3a | GGATTAA(2-Deoxyuridine)AATGGAAGAGACTTTCGTG | pYeDP60u |
| 50 | RV_pSc483_BnaA03.BOR1;3a | GGGTTAA(2-Deoxyuridine)TCAATGAGAGCCCTGGTTC | pYeDP60u |
| 51 | FW_pSc484_BnaA05.BOR1;2a | GGATTAA(2-Deoxyuridine)AATGGAAGAGACGTTTGTGCCG | pYeDP60u |
| 52 | RV_pSc484_BnaA05.BOR1;2a | GGGTTAA(2-Deoxyuridine)TCACTTTGACGAGGACTGGTTC | pYeDP60u |
| 53 | FW_pSc485_BnaC04.BOR1;2c | GGATTAA(2-Deoxyuridine)AATGGAAGAGACGTTTGTGCCG | pYeDP60u |
| 54 | RV_pSc485_BnaC04.BOR1;2c | GGGTTAA(2-Deoxyuridine)TCAGTTGAGCGAGGACTGG | pYeDP60u |
| 55 | FW_pSc486_BnaA04.BOR1;1a | GGATTAA(2-Deoxyuridine)AATGGAAGAGACGTTTGTGCCG | pYeDP60u |
| 56 | RV_pSc486_BnaA04.BOR1;1a | GGGTTAA(2-Deoxyuridine)TCAGTTGAGCGAGGACTGG | pYeDP60u |
| 57 | FW_pSc487_BnaC04.BOR1;1c | GGATTAA(2-Deoxyuridine)AATGGAAGAGACCTTTGTGCCG | pYeDP60u |
| 58 | RV_pSc487_BnaC04.BOR1;1c | GGGTTAA(2-Deoxyuridine)TCAGTTGAACGAGGACTGG | pYeDP60u |

**RT-PCR-Primer-Table: Primers which have been used in this study for RT-PCR:**

| Nr. | Primer name | Sequence | Target gene |
| --- | --- | --- | --- |
| 59 | FW_EX_AtNIP5;1 | CACCGATTTTCCCTCTCCTGAT | AtNIP5;1 |
| 60 | RV_EX_AtNIP5;1 | GCATGCAGCGTTACCGATTA | AtNIP5;1 |
| 61 | FW_EX_AtEF1α | CCTTGGTGTCAAGCAGATGA | AtEF1α |
| 62 | RV_EX_AtEF1α | TGAAGACACCTCCTTGATGATTT | AtEF1α |
| 63 | FW_EX_PL98_BnaC05.NIP7;1a | gagagactcgagATGAATGTTGAGGTACGGT | BnaC05.NIP7;1a |
| 64 | RV_EX_PL98_BnaC05.NIP7;1a | ctctctggcgcgccTTAGCGTAACAGGGAAGAG | BnaC05.NIP7;1a |
| 65 | FW_EX_PL97_BnaC06.NIP6;1a | gagagactcgagATGGATCACGAGGAAATACC | BnaC06.NIP6;1a |
| 66 | RV_EX_PL97_BnaC06.NIP6;1a | ctctctggcgcgccTCATCTCCTGAAACTCCTCTTCTC | BnaC06.NIP6;1a |
| 67 | FW_EX_PL95_LOC106388529 | gagagactcgagATGTCTCCGCCAGAGGCTGAAATG | LOC106388529 |
| 68 | RV_EX_PL95_LOC106388529 | ctctctggcgcgccTTAACGACGGAAGCTTCTAACCTGACGCGGTG | LOC106388529 |
| 69 | FW_EX_PL99_BnaC04.NIP4;1b | ACTTGCGGGGTCCTTACTAGCT | BnaC04.NIP4;1b |
| 70 | RV_EX_PL99_BnaC04.NIP4;1b | CTATCCGTGGCAACACCAGAT | BnaC04.NIP4;1b |
| 71 | FW_EX_PL100_BnaC04.NIP4;1a | TCTGATTCAGCGGCACAAGCA | BnaC04.NIP4;1a |
| 72 | RV_EX_PL100_BnaC04.NIP4;1a | GGATTCATCGATGCTCCG | BnaC04.NIP4;1a |

**Promoter-Table: Amplified *BnaNIP5;1* promoter sequences**

| Gene name | Chromosome* | Strand | Start* | End* | Size in bp |
| --- | --- | --- | --- | --- | --- |
| BnaC02.NIP5;1a | chrA02 | + | 28885124 | 28887508 | 2385 |
| BnaA07.NIP5;1c | chrA07 | + | 13837830 | 13840225 | 2396 |
| BnaA03.NIP5;1b | chrA03 | - | 11722230 | 11724546 | 2317 |

* Genoscope (http://www.genoscope.cns.fr/ brassicanapus/)

**qRT-PCR-Table: Primers which have been used for the qRT-PCR**

| **‘Reference’ genes** | | |
| --- | --- | --- |
| **Gene symbol** | **Primer pair**  **(forward/reverse)** | **Comment** |
| EXP | FW_GATGAATATCCTCCTGATGCTAACC  RV_CTTGCATGATGATCAGGAAAGC | - |
| EF1a | FW_AAGTACGCATGGGTCTTGGA  RV_GGTGGTCTCGAACTTCCAGA | - |
| TIP41 | FW_TGAAGAGCAGATTGATTTGGCT  RV_ACACTCCATTGTCAGCCAGTT | - |
| **‘Nodulin26-like Intrinsic Protein’ genes** | | |
| **Gene name** | **Primer pair**  **[forward (FW_) / reverse (RV_)]** | **Comment** |
| BnaC05.NIP3;1b | FW_GGGACATTCTCTCTTGTATTTGCC  RV_GGAGACATGACCAATTGAGTAGC | - |
| BnaA05.NIP3;1b | FW_GGACATTCTCTCTTGTATTTGCG  RV_GGAGACATGACCAATTGAGTAGG | - |
| BnaA05.NIP3;1a | FW_CGATAGAATCTCTTCGGCTCTTG  RV_TACTACAGTTGCACCAATTGCAAGA | - |
| BnaC05.NIP3;1a | FW_TCGGCAATCGTGGTAAATGATACA  RV_ATCGAGTAGGTCATAACCATTACGC | - |
| BnaA08.NIP3;1c | FW_TGGATCATTCGCAGGCATTGC  RV_GTAGACCGTAACATATCATAAGTCCAT | Primer pair is only specific in root tissue |
| BnaC08.NIP3;1c | FW_TGGATCATTCGCAGGCATTGC  RV_GTAGACCGTAACATATCATAAGTCCAC | Primer pair is only specific in root tissue |
| BnaA04.NIP4;1a | FW_CCAGCAGCTTACTTTGGAACAAT  RV_TGCTGGATTCATCGATGCTCCC | - |
| BnaA04.NIP4;1b | FW_CCGGCCGTCACCTTAACTT  RV_ATTCTCCAATCGCGCGACT | Specific primer pair, but efficiency at ~80 % |
| BnaC04.NIP4;1a | FW_TCTGATTCAGCGGCACAAGCA  RV_GGATTCATCGATGCTCCG | - |
| BnaC04.NIP4;1c | FW_TTGTATAATATGTTTCGGG  RV_TAACTCCGGCGATGGGACCT | - |
| BnaC04.NIP4;1b | FW_ACTTGCGGGGTCCTTACTAGCT  RV_CTATCCGTGGCAACACCAGAT | - |
| BnaC06.NIP4;2a | FW_CTTGCGAGTTTGATACTT  RV_TCGCACGACTATCAGTGGCG | - |
| BnaA03.NIP5;1b | FW_TTGCCGGTGATGGGAAACCG  RV_GAACTCGGCCCCGAGCTTT | - |
| BnaA02.NIP5;1a | FW_TGCTTGCCGGTGATGGGACACA  RV_GTTTAAGTGAGCCCCTGAG | - |
| BnaA07.NIP5;1c | FW_ACGACACGTGCTTCACCGATA  RV_AGGAACAGTCACACCTCCT | - |
| BnaC02.NIP5;1a | FW_AATTGGTAACGCGGCATGT  RV_GTTTAAGTGAGCTCCTGAA | - |
| BnaC06.NIP5;1c | FW_AATGGGTGCGGTGGCGGTA  RV_AGCTTGCGAGTAAGGGAGG | - |
| BnaC03.NIP5;1b | FW_TACCTGCTTACATAGCAGCG  RV_ATTCATAGATGCACCAGTA | - |
| BnaA07.NIP6;1b | FW_CGGGGCACATCTCAACCCT  RV_ATTCATTGACGCAGAAGTG | - |
| BnaA02.NIP6;1a | FW_TCTCTCCCTGCAATGGACCA  RV_CAGAAGTGACTTTGGTGTGT | - |
| BnaA02.NIP6;1c | FW_TAGATACACACCAAAGTCG  RV_ACTTACGGTAGAGGGAGAT | - |
| BnaC06.NIP6;1a | FW_TCTTTCTCTCCAATATGGATC  RV_TGTCCATTCCTCTTTCCTTC | Primer pair amplifies BnaC06.NIP6;1a & BnaA07.NIP6;1b |
| BnaA05.NIP7;1a | FW_TCATTAGCAGTACTCAGTTG  RV_ACTACGACAACCACCGATAGA | - |
| BnaC05.NIP7;1a | FW_TCATAAGCAGTACTCAGTTA  RV_ACTACAACAACCACCGATAGT | - |
| **‘BOR1’ genes** | | |
| **Gene name** | **Primer pair**  **[forward (FW_) / reverse (RV_)]** | **Reference** |
| BnaC04.BOR1;1c | FW_CCTTTAATCAATCCCCACTATCAAAGATTTG  RV_GCCAAGAAGAGGTCTCGTCCAAGTTCAGAC | Chen et al., 2018 |
| BnaA04.BOR1;1a | FW_ACCAATTCTGGGAGAGAATCC  RV_CCTGCGATTGGGATCCAC |  |
| BnaA05.BOR1;2a | FW_GTTTAAAAGAACTGAAAGAATCAACG  RV_CAGCAACGCATCCTCCC |  |
| BnaC04.BOR1;2c | FW_CATCACATGCAGACACCATTG  RV_CTACCATTGTGGACTGAAGGAG |  |
| BnaA03.BOR1;3a | FW_ACTTCGTATCCAGGAGACTCG  RV_CTAAACACTTGAGACAAACTCCTAC |  |
| BnaC03.BOR1;3c | FW_TGCAACGTTCACTATTTTCCAG  RV_GCTGCGAGATTGAAGGGTAAC |  |

**References - Supporting Experimental Procedures**

**Chen, H., Zhang, Q., He, M., Wang, S., Shi, L. and Xu, F.** (2018) Molecular characterization of the genome-wide BOR transporter gene family and genetic analysis of BnaC04.BOR1;1c in *Brassica napus*. *BMC Plant Biol*. 18, 193. https://doi.org/10.1186/s12870-018-1407-1

**Clough, S.J. and Bent, A.F.** (1998) Floral dip: a simplified method for Agrobacterium‐mediated transformation of *Arabidopsis thaliana*. *Plant J*. 16, 735-743. https://doi.org/10.1046/j.1365-313x.1998.00343.x

**Drummond, A.J. and Rambaut, A.** (2007) BEAST: Bayesian evolutionary analysis by sampling trees. *BMC Evol. Biol*. 7, 214. https://doi.org/10.1186/1471-2148-7-214

**Eggert, K. and von Wirén, N.** (2013) Dynamics and partitioning of the ionome in seeds and germinating seedlings of winter oilseed rape. *Metallomics*, 5, 1316-1325. https://doi.org/10.1039/C3MT00109A

**Fetter, K., Van Wilder, V., Moshelion, M. and Chaumont, F.** (2004) Interactions between plasma membrane aquaporins modulate their water channel activity. *Plant Cell*, 16, 215-228. https://doi.org/10.1105/tpc.017194

**Heniko, S. and Heniko, J.G.** (1992) Amino acid substitution matrices from protein blocks. *Proc. Natl. Acad. Sci. USA*, 89, 10915-10919. https://doi.org/10.1073/pnas.89.22.10915

**Katoh, K. and Standley, D.M.** (2013) MAFFT multiple sequence alignment software version 7: Improvements in performance and usability. *Mol. Biol. Evol*. 30, 772-780. https://doi.org/10.1093/molbev/mst010

**Kearse, M., Moir, R., Wilson, A., Stones-Havas, S., Cheung, M., Sturrock, S., Buxton, S., Cooper, A., Markowitz, S., Duran, C., Thierer, T., Ashton, B., Meintjes, P. and Drummond, A.** (2012) Geneious Basic: an integrated and extendable desktop software platform for the organization and analysis of sequence data. *Bioinformatics*, 28,1647-1649. https://doi.org/10.1093/bioinformatics/bts199

**Murashige, T. and Skoog, F.** (1962) A revised medium for rapid growth and bio assays with tobacco tissue cultures. *Physiol. Plantarum*, 15, 473-497. https://doi.org/10.1111/j.1399-3054.1962.tb08052.x

**Nakagawa, T., Kurose, T., Hino, T., Tanaka, K., Kawamukai, M., Niwa, Y., Toyooka, K., Matsuoka, K., Jinbo, T. and Kimura, T.** (2007) Development of series of gateway binary vectors, pGWBs, for realizing efficient construction of fusion genes for plant transformation. *J. Biosci. Bioeng*. 104, 34-41. https://doi.org/10.1263/jbb.104.34

**Nour-Eldin, H.H., Hansen, B.G., Nørholm, M.H., Jensen, J.K. and Halkier, B.A.** (2006) Advancing uracil-excision based cloning towards an ideal technique for cloning PCR fragments. *Nucleic Acids Res*. 34, e122. https://doi.org/10.1093/nar/gkl635

**Opperdoes, F.B.** (2003) Phylogenetic analysis using protein sequences. In *The Phylogenetic Handbook: A Practical Approach to Phylogenetic Analysis and Hypothesis Testing* (Lemey, F., Salemi, M. and Vandamme, A-M., eds). Cambridge: University Press, pp. 210-266.

**Pommerrenig, B., Junker, A., Abreu, I., Bieber, A., Fuge, J., Willner, E., Bienert, M.D., Altmann, T. and Bienert, G.P.** (2018) Identification of rapeseed (*Brassica napus*) cultivars with a high tolerance to boron-deficient conditions. *Front. Plant Sci*. 9, 1142. https://doi.org/10.3389/fpls.2018.01142

**Rambaut, A. and Drummond, A.** (2007) TRACER, Vo1. 5. Available at: http://beast.bio.ed.ac.uk/Tracer [accessed June 11, 2011]

**Ronquist, F. and Huelsenbeck, J.P.** (2003). MrBayes 3: Bayesian phylogenetic inference under mixed models. *Bioinformatics*, 19, 1572-1574. https://doi.org/10.1093/bioinformatics/btg180

### Schmutzer, T., Samans, B., Dyrszka, E., Ulpinnis, C., Weise, S., Stengel, D., Colmsee, C., Lespinasse, D., Micic, Z., Abel, S., Duchscherer, P., Breuer, F., Abbadi, A., Leckband, G., Snowdon, R. and Scholz, U. (2015) Species-wide genome sequence and nucleotide polymorphisms from the model allopolyploid plant *Brassica napus*. *Sci. Data*, 2, 150072. https://doi.org/10.1038/sdata.2015.72

**Van der Fits, L., Deakin, E.A., Hoge, J.H.C. and Memelink, J.** (2000) The ternary transformation system: constitutive virG on a compatible plasmid dramatically increases Agrobacterium-mediated plant transformation. *Plant Mol. Biol*. 43, 495-502. https://doi.org/10.1023/A:1006440221718

**Vandesompele, J., De Preter, K., Pattyn, F., Poppe, B., Van Roy, N., De Paepe, A. and Speleman, F.** (2002) Accurate normalization of real-time quantitative RT-PCR data by geometric averaging of multiple internal control genes. *Genome Biol.* 3, RESEARCH0034. https://doi.org/10.1186/gb-2002-3-7-research0034
