# Supplementary material for: Boron demanding tissues of Brassica napus express specific sets of functional Nodulin26‐like Intrinsic Proteins and BOR1 transporters
Source: Plant J. 2019 Jul 15;100(1):68–82. doi: 10.1111/tpj.14428 (PMC6852077; doi:10.1111/tpj.14428)
Supplement: Supplementary file 5 [file TPJ-100-68-s005.docx]

**Figure S1**: **Tissue** **boron concentrations of *Brassica napus* *cv. PBY018* plants grown under B-sufficient or B-deficient growth conditions at the reproductive stage. Related to Figure 5 and 6.**

Phenotype of *B. napus* flowers grown under B-sufficient (a) and B-deficient (b and c) growth conditions. B-deficient flowers in (b) will develop within about a week to an inflorescence similar to what is displayed in (c). Boron concentrations of indicated *B. napus* tissues have been determined by ICP-MS (d). Chart bars represent the average ± SD of tissues from three plants (n = 3). Asterisks indicate significant differences (*** p < 0.001, *t*-test) of the recorded parameter between B-deficient and B-sufficient growth conditions (Ns = not significant).

**Figure S2: Minimum open reading frames (ATG-Stop) upstream of Brassica napus NIP5;1 genes.**

(a) Alignment of the upstream regions (350 nucleotides) of the coding sequences of all six B. napus NIP5;1 genes. The minimum open reading frames (ATG-Stop) are displayed in bold, underlined and blue letters. (b) Table describing the nucleotide sequence and the position of the minimum open reading frames being located upstream of the coding sequences of all six B. napus Darmor-bzh BnaNIP5;1 genes.

**Figure S3:** ***BnaNIP5;1* promoters are upregulated in Arabidopsis roots and shoots under B limitation**

B-dependent *BnaNIP5;1* promoter activity in Arabidopsis roots and shoots visualized by promoter GUS analysis. Homozygous T3 *BnaNIP5;1_pro_:GUS* transgenic plant lines were grown for 7 days without the supply of any boric acid and then transferred to medium containing 0.1 (B-deficient) or 100 (B-sufficient) µM boric acid. After 9 days, GUS staining was performed for 4 hours (a) or 8 hours (b). Representative shoots, primary roots (PR), lateral roots (LR) and lateral root branch point (LR branch) pictures are displayed. Black arrowheads indicate differently intense GUS signals in roots. For each *BnaNIP5;1_pro_:GUS* construct two independent transgenic lines have been assessed with identical results. Scale bars: shoots = 2 mm and roots = 0.5 mm.

**Methods S1:** **Supporting Experimental Procedures.**
